# Physician-defined severe toxicities occurring during and after cancer treatment: Modified consensus definitions and clinical applicability in the evaluation of cancer treatment

**DOI:** 10.3389/fped.2023.1155449

**Published:** 2023-04-26

**Authors:** Camilla Grud Nielsen, Birthe Lykke Thomsen, Bodil Als-Nielsen, Rachel Conyers, Sima Jeha, Marion K. Mateos, Wojciech Mlynarski, Rob Pieters, Mathias Rathe, Kjeld Schmiegelow, Liv Andrés-Jensen

**Affiliations:** ^1^ Department of Pediatrics and Adolescent Medicine, Copenhagen University Hospital, Copenhagen, Denmark; ^2^ Section of Biostatistics, University of Copenhagen, Copenhagen, Denmark; ^3^ Department of Paediatrics, University of Melbourne, Melbourne, VIC, Australia; ^4^ Pharmacogenomics, Stem Cell Biology, Murdoch Children’s Research Institute, Melbourne, VIC, Australia; ^5^ Children’s Cancer Centre, The Royal Children’s Hospital, Melbourne, VIC, Australia; ^6^ Department of Oncology, St Jude Children’s Research Hospital, Memphis, TN, United States; ^7^ Kids Cancer Centre, Sydney Children’s Hospital Randwick, Sydney, NSW, Australia; ^8^ Discipline of Paediatrics and Child Health, School of Clinical Medicine, UNSW Medicine & Health, UNSW, Sydney, NSW, Australia; ^9^ Children’s Cancer Institute, Lowy Cancer Research Centre, UNSW, Sydney, NSW, Australia; ^10^ Department of Pediatrics, Oncology & Hematology, Medical University of Lodz, Lodz, Poland; ^11^ Princess Maxima Center for Pediatric Oncology, Utrecht, Netherlands; ^12^ Department of Pediatric Hematology and Oncology, Hans Christian Andersen’s Children’s Hospital, Odense University Hospital, Odense, Denmark; ^13^ Department of Clinical Research, University of Southern Denmark, Odense, Denmark

**Keywords:** cancer treatment, toxicities, childhood cancer, long-term, severe-toxicity-free-survival

## Abstract

Overall survival after cancer is increasing for the majority of cancer types, but survivors can be burdened lifelong by treatment-related severe toxicities. Integration of long-term toxicities in treatment evaluation is not least important for children and young adults with cancers with high survival probability. We present modified consensus definitions of 21 previously published physician-defined Severe Toxicities (STs), each reflecting the most serious long-term treatment-related toxicities and representing an unacceptable price for cure. Applying the Severe Toxicity (ST) concept to real-world data required careful adjustments of the original consensus definitions, translating them into standardized endpoints for evaluating treatment-related outcomes to ensure that (1) the STs can be classified uniformly and prospectively across different cohorts, and (2) the ST definitions allow for valid statistical analyses. The current paper presents the resulting modified consensus definitions of the 21 STs proposed to be included in outcome reporting of cancer treatment.

## 1. Introduction

Overall survival probability after a cancer diagnosis is increasing for the majority of cancer types. This is not least the case for most childhood cancers, where acute lymphoblastic leukemia (ALL) accounting for 25% of all cases (1) now has a five-year overall survival probability that exceeds 90% with the best contemporary therapy (2). However, the high survival probability comes at a price, since a significant proportion of the growing population of survivors is burdened by lifelong treatment-related toxicities, some of which are disabling or even life-threatening (3–6). Hence, traditional treatment outcome measures like overall survival and cancer-related event-free survival, i.e., survival without experiencing resistant disease, relapse, or second malignant neoplasms, have become insufficient since they fail to account for the burden of cancer therapy caused by treatment-related toxicities. This may reflect the lack of internationally approved, standardized definitions and data-capturing strategies for such treatment-related long-term serious toxicities.

Recently, ALL consortia across Europe, the USA, Asia, and Australia initiated a project addressing this issue, focusing on physician-defined severe toxicities (STs) (7). The project aimed to define and subsequently capture the most severe long-term treatment-related toxicities in an expert consensus-based manner. The purpose was to enable future integration of these toxicities in outcome evaluation alongside the overall survival and the cancer-related event-free survival for a more comprehensive evaluation of treatment protocols (7), a particularly important aspect for childhood cancers with high survival probability.

This international initiative resulted in consensus definitions of 21 STs that were selected and defined based on five generic criteria (not present before cancer diagnosis; symptomatic; objective; of unacceptable severity; and permanent or requiring unacceptable treatments). Each of the STs was considered an unacceptable trade-off for cure, i.e., the conditions are of such severity that had the condition in question been predictable at cancer diagnosis, it would likely have led to a modification in cancer therapy.

This international consensus on selection and definition of these 21 STs was the first step towards integrating the most severe treatment-related toxicities as a new standard in cancer treatment evaluation. However, during the subsequent design phase of an international evaluation of the occurrence of STs across five childhood ALL cohorts from Australia, Europe, and the US, it became clear that further refinements of the definitions were necessary to allow uniform application, valid statistical analyses, and to ensure that the Severe Toxicity (ST) definitions can be applied prospectively.

Evaluation of the occurrence of STs involves several relevant population summary measures to supplement the overall survival and the cancer-related event-free survival. Additional outcomes to be considered are (1) severe-toxicity-free-survival (STFS), which includes the STs in the event definition, (2) the cumulated probability of experiencing each of the STs, and (3) the burden of experiencing multiple STs. All these measures require a time-to-event approach for the statistical analyses.

Although the original consensus definitions were clinically relevant conditions representing an unacceptable price for cure, the definitions were not designed in a way allowing for valid statistical inference. An example of a major challenge is the generic criterion that an ST should be *permanent* or only correctable by an unacceptable treatment. Conditioning on the future leads to invalid statistical analyses of time-to-event data (8), hence, the use of the term “permanent” is problematic and the corresponding clinical issue needed to be addressed in an appropriate way. To avoid conditioning on the future, it was also necessary to make analytically valid definitions of the timing of each of the STs. Avoiding conditioning on the future is important to ensure the clinical applicability of the concept in the ongoing surveillance of patients. Due to these issues, an updated version of the ST definitions was required.

This paper describes the identified problems and challenges, the principles behind the suggested solutions, as well as the final, modified consensus definitions of 21 STs obtained through a Delphi process. The revised ST definitions enable uniform future reporting of STs across international cohorts as an integrated part of cancer treatment evaluation thereby paving the way for research in the field.

## 2. Methods

### 2.1. Identification of challenges and problems with original ST definitions

**Figure 1:**
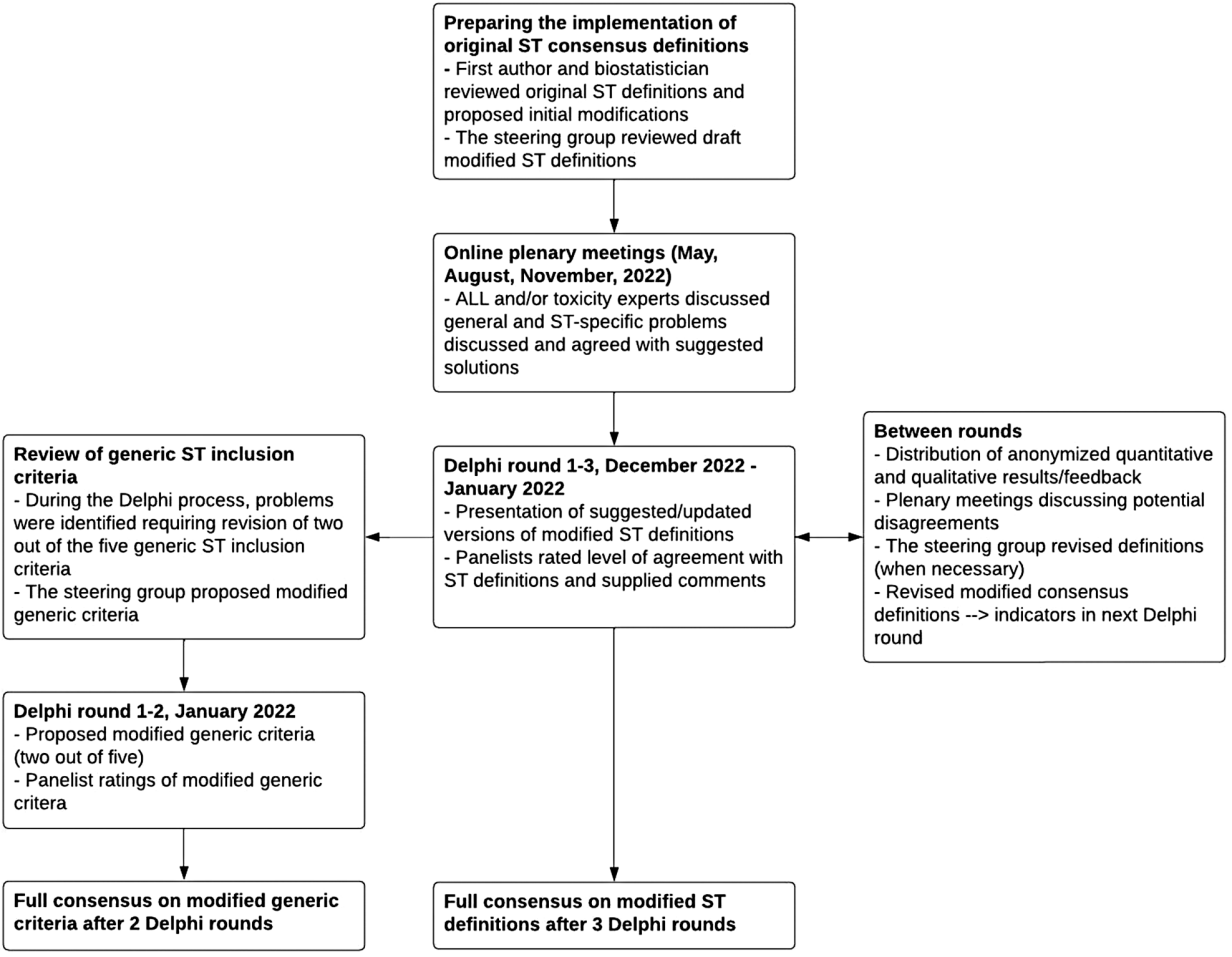
Process leading to modified ST definitions and modified generic ST inclusion criteria.

During the planning phase of the international validation of the STFS measure, general problems and challenges of the original consensus definitions as well as ST-specific issues were identified in collaboration with a biostatistician with expertise in time-to-event analyses. These problems and suggestions for solutions were presented and discussed at online plenary meetings including principal investigators representing the cohorts participating in the planned international evaluation of the STs. Based on the discussions, modified ST definitions were proposed, and these modified ST definitions were reviewed in a Delphi process (Figure 1).

### 2.2. Delphi process to obtain modified ST definitions

The aim of the Delphi process was to establish consensus on the 21 modified ST definitions. Panelists included ALL and/or toxicity experts and a biostatistician with expertise in time-to-event analyses. Panelists were asked to anonymously rate each definition by stating their level of agreement as either high, medium, or low, and were required to leave a comment with suggestions for improvement if indicating medium or low level of agreement. Between each round, panelists received quantitative feedback on the level of agreement and qualitative feedback on all received comments. Online plenary meetings were held allowing for clarification of potential disagreements. Consensus was defined *a priori* as requiring 100% consensus.

## 3. Results

### 3.1. Challenges and problems with original ST definitions

During the planning phase of the international evaluation of the occurrence of STs, seven general challenges with the original ST definitions were identified: (1) the use of the term “*permanent*”, (2) including only conditions occurring *during* treatment for some STs, (3) the use of the term “*planned*”, (4) evaluation of activities of daily living (ADL), (5) lack of standardized evaluation (6) conditions likely to be temporarily affected during cancer therapy, and (7) the presence of pre-existing conditions that precludes a specific ST or increases the probability of developing a specific ST. Each of these seven general challenges are described in detail below, presenting the identified problems and their solutions. The modified generic criteria are presented in Table 1 and the modified ST definitions are presented in Table 2 (original generic criteria and original ST definitions are available in the Supplementary Material). Furthermore, some additional ST-specific aspects were addressed (available in Supplementary Material). The results from each Delphi round and changes and arguments for each toxicity are available in the Supplementary Material.

**Table 1:** Modified generic inclusion criteria.

**Not present before cancer diagnosis** Pre-existing conditions cannot be considered treatment-related toxicity. For each toxicity, all relevant known pre-existing conditions must be registered to allow for valid statistical analyses (it is not required to screen for such conditions). Relevant pre-existing conditions may either • increase the probability of having a severe toxicity classified (e.g., being blind in one eye so only the other eye needs to be affected during treatment; or having Li-Fraumeni syndrome, relevant for developing second malignant cancer), or • preclude the possibility of a classification as severe toxicity for the patient (e.g., being blind in both eyes before cancer diagnosis)
**Symptomatic** To ensure equal probability of capturing the condition across different protocols using different screening strategies, the condition must be symptomatic and expected to lead to a clinical diagnosis without use of routine screening. • Compensated cardiac failure detected by routine echocardiogram is not included, whereas severe, symptomatic cardiac failure is included.
**Objective** The condition must be uniformly classifiable across different patients and by different observers. • Chronic pain, nausea, or fatigue, which are subjective, are not included, although these conditions can represent a substantial burden to the survivor.
**Unacceptable severity** The condition must be so severe, that it is considered an unacceptable tradeoff for disease control—i.e., had the condition been predictable at cancer diagnosis, it would probably have led to a change in anticancer therapy. • Physical and mental conditions that substantially affect self-care and instrumental activities of daily living or posing substantial threat of early mortality fulfill this criterion. • This consideration mirrors current actions (e.g., as reduction of anthracycline use in patients with Down Syndrome, reduction of thiopurine doses in patients with TPMT deficiency) or concerns related to re-exposure after severe drug-induced toxicity (e.g., re-exposure to asparaginase following asparaginase associated pancreatitis).
**Persisting severity or correctable only by unacceptable treatments** The condition must have been present for a sustained period or be corrected by a treatment, which itself is considered unacceptable. • Acute events are not included, but sequelae such as severe cognitive deficits following cerebral hemorrhage or amputation of a limb following severe infections, are. • Organ transplantation is an example of an unacceptable treatment since it is itself associated with risk of severe mortality and morbidity, whereas growth hormone replacement is an example of a treatment that is not considered unacceptable.

**Table 2:** Modified consensus definitions of the 21 severe toxicities.

Severe toxicity (ST)	Consensus definition, time of ST and additional notes
Hearing loss	**Definition:** Persisting bilateral hearing loss emerging during or after anticancer therapy and defined as >40 dB hearing loss at ≤2 kHz that persists for ≥12 months or requiring cochlear implant. **Time of ST:** Date when at least one of the criteria are met for both ears, i.e., both ears having audiometry identified >40 dB hearing loss at ≤2 kHz that has persisted for ≥12 months and/or cochlear implant surgery (whichever occurs first).
Blindness	**Definition:** Untreatable blindness emerging during or after anticancer therapy, defined as visual acuity of <20/200 or a corresponding visual field loss to <10° in the stronger eye with the best possible correction. **Time of ST:** Date when blindness is identified as visual acuity of <20/200 or a corresponding visual field loss <10° in the stronger eye with the best possible correction.
Heart failure	**Definition:** Persisting (≥12 months), symptomatic cardiac dysfunction emerging during or after anticancer therapy and defined by a decrease in left ventricular ejection fraction to a value <40% or fractional shortening to <20% and one of the following: (i) age 0–1 years: marked tachypnoea or diaphoresis with feeding or prolonged feeding times with growth failure or tachypnoea, retractions, grunting, or diaphoresis at rest^a^, (ii) age 1–17.9 years: marked dyspnea on exertion or at rest^a^, or age ≥18 years: marked dyspnea, palpitations or anginal pain on exertion or at rest^b^; or symptomatic cardiac dysfunction requiring heart transplantation. **Time of ST:** Date when the condition fulfilling the clinical *and* paraclinical criteria has persisted for 12 months, or when the patient is referred for transplantation (whichever occurs first). **Additional notes:** Screening of all patients with echocardiographic measures is not required, but echocardiographic confirmation is required for inclusion as ST. Echocardiographic measures are provided because international surveillance guidelines accept its use as the primary surveillance tool for cardiotoxicity. A repeat echocardiogram is expected to be done at least 1 week apart to confirm cardiac dysfunction.
Coronary artery disease	**Definition:** Coronary artery disease emerging during or after anticancer therapy and resulting in myocardial infarction, or requiring angioplasty (balloon or stent), or coronary bypass surgery. **Time of ST:** Date of myocardial infarction or when angioplasty (balloon or stent) or coronary bypass surgery is performed (whichever occurs first).
Arrhythmia	**Definition:** Arrhythmia emerging during or after anticancer therapy, requiring a pacemaker or an implantable cardioverter defibrillator. **Time of ST:** Date when pacemaker or implantable cardiac defibrillator is implanted.
Heart valve disease	**Definition:** Heart valve dysfunction emerging during or after anticancer therapy and requiring surgical valve replacement. **Time of ST:** Date when surgical valve replacement is performed.
Gastrointestinal failure	**Definition:** Gastrointestinal failure emerging during or after anticancer therapy, resulting in persistently (≥12 months) requiring parenteral nutrition, or placement of a PEG tube due to physical inability to eat or swallow that persists for ≥12 months, or placement of a stoma that persists for ≥12 months. **Time of ST:** Date when the condition fulfilling the clinical criteria in the ST definition has persisted for 12 months.
Hepatic failure	**Definition:** Severe and persisting (≥12 months) hepatobiliary failure emerging during or after anticancer therapy, and defined as any of the following: symptomatic, decompensated liver disease including cirrhosis and portal hypertension that is not responsive to pharmacologic and endoscopic management; or any hepatobiliary failure requiring liver transplantation. **Time of ST:** Date when the condition fulfilling the clinical criteria in the ST definition has persisted for 12 months or when the patient is referred for transplantation (whichever occurs first). **Additional notes:** Typical symptoms of hepatic failure include fatigue, gum bleeding, epistaxis, itching, and icterus in all age groups in addition to impaired growth and delayed puberty in children. Patients who undergo a portosystemic shunt for hepatic disease are included in this definition because shunts are usually reserved for refractory disease, which may serve as a bridge to liver transplant.
Insulin dependent diabetes	**Definition:** Persisting (≥12 months) insulin dependent diabetes emerging during or after anticancer therapy. **Time of ST:** Date when the condition fulfilling the clinical criteria in the ST definition has persisted for 12 months. **Additional notes:** Insulin dependent diabetes is treatable; however, is included because of substantial risk of cardiovascular disease and end-organ failure.
Renal failure	**Definition:** Persisting (≥12 months) loss of kidney function emerging during or after anticancer therapy that requires dialysis or renal transplantation. **Time of ST:** Date when the condition fulfilling the clinical criteria in the ST definition has persisted for 12 months or when the patient is referred for transplantation (whichever occurs first).
Pulmonary failure	**Definition:** Chronic lung failure (including pulmonary fibrosis and bronchiolitis obliterans) emerging during or after anticancer therapy and requiring daily oxygen supplement (≥12 months) or lung transplantation. **Time of ST:** Date when the condition fulfilling the clinical criteria in the ST definition has persisted for 12 months or when the patient is referred for transplantation (whichever occurs first).
Osteonecrosis	**Definition:** Osteonecrosis occurring during or after anticancer therapy and requiring total joint arthroplasty; or resulting in grade 4 toxicity according to the Ponte di Legno Toxicity Working Group Criteria (i.e., symptomatic with deformation by imaging of one or more joints or substantially affecting self-care ADL* for ≥12 months). *E.g., requiring daily assistance beyond what is considered age-appropriate with at least one self-care ADL and/or requiring instrumental aid, such as wheelchair or walking stick, for mobility. **Time of ST:** Date when the condition fulfilling the clinical criteria in the ST definition has persisted for 12 months or when total joint arthroplasty is performed (whichever occurs first). **Additional notes:** Examples of self-care ADL include grooming/personal hygiene, dressing, toileting/continence, transferring/ambulating, and eating.
Amputation and physical deformation	**Definition:** Amputation of extremities, severe spinal deformation, and disabling scleroderma, scarring, or contractions affecting self-care ADL substantially* for ≥12 months or causing substantial facial disfigurement, and defined as follows: lower limb amputation (proximal to ankle), upper limb amputation (proximal to wrist), scoliosis, kyphosis, or lordosis affecting self-care ADL substantially, scarring or contractions affecting range of movement that affects self-care ADL substantially, scleroderma affecting self-care ADL substantially, amputation of nose, amputation of one or both eyes, complete facial palsy (unilateral- or bilateral). Conditions emerging during or after anticancer therapy are included. *E.g., requiring daily assistance beyond what is considered age-appropriate with at least one self-care ADL and/or requiring instrumental aid, such as a wheelchair or walking stick, for mobility. **Time of ST:** Date when amputation is performed, or when one of the following conditions fulfilling the clinical criteria in the ST definition has persisted for ≥12 months: scoliosis, kyphosis, lordosis, scarring or contractions, or scleroderma, or when diagnosed with complete facial palsy (unilateral or bilateral). **Additional notes:** Examples of self-care ADL include grooming/personal hygiene, dressing, toileting/continence, transferring/ambulating, and eating.
Cognitive dysfunction	**Definition:** Any substantial impairment of neurocognitive functions* emerging during or after anticancer therapy, that affects instrumental ADL substantially** and persists for ≥12 months *after* ending anticancer therapy. *E.g., executive function (planning and organization), sustained attention, memory (particularly visual sequencing, temporal memory), processing speed, visual-motor integration, fine motor dexterity, diminished performance on IQ-tests, and learning deficits. **E.g., severely restricted participation in school, vocational training, practice, and career, and/or requiring daily assistance beyond what is considered age-appropriate with other key activities of instrumental ADL. **Time of ST:** Date when the condition fulfilling the clinical criteria in the ST definition has persisted for 12 months in the period *after* ending anticancer therapy. **Additional notes:** Cognitive dysfunction emerging after ending anticancer therapy must persist for ≥12 months to be classified as an ST. Pre-existing conditions relevant for this condition include not having met normal developmental milestones and/or evidence of developmental delay at the time of diagnosis. Other examples of instrumental ADL include cooking, cleaning, managing finances, managing medications. “*Verified” cognitive dysfunction is defined as:* The patient lives in an institution due to cognitive dysfunction and/or the patient requires daily assistance with instrumental ADL due to cognitive dysfunction. *“Possible” cognitive dysfunction is defined as:* The patient scores below normal range (<2.5 percentile) in neuropsychological tests^§^, but the degree of impact on instrumental ADL is uncertain and/or the patient has substantially impaired instrumental ADL, but it is uncertain if it depends on this specific condition or is due to other reasons, e.g., psychiatric disease. § Not specified due to variety in instruments used across centers. Neuropsychological testing is likely to be performed in patients with severe cognitive dysfunction but is not required for inclusion in the STFS measure.
Seizures	**Definition:** Seizures emerging during or after anticancer therapy that require neurosurgical intervention to reach seizure control, or that fulfil the International League Against Epilepsy definition for drug-resistant epilepsy [“defined as failure of adequate trials of two tolerated and appropriately chosen and used anti-epileptic drug schedules (whether as monotherapies or in combination) to reach sustained seizure freedom”]^c^. **Time of ST:** Date when the patient experiences a seizure despite adequate trials of two tolerated and appropriately chosen and used anti-epileptic drug schedules, or when neurosurgery is performed to reach seizure control (whichever occurs first).
Psychiatric disease	**Definition:** Any psychiatric disorder emerging during or after anticancer therapy, that is severe enough to require mental health input (psychology or psychiatry), and affects instrumental ADL substantially* and persists for ≥12 months *after* ending anticancer therapy. *E.g., severely restricted participation in school, vocational training, practice, and career, and/or requiring daily assistance beyond what is considered age-appropriate with other key activities of instrumental ADL. **Time of ST:** Date when the condition fulfilling the clinical criteria in the ST definition has persisted for 12 months *after* ending anticancer therapy. **Additional notes:** As evaluated by the physician because uniform and objective evaluation is not done across study groups. Other examples of instrumental ADL include cooking, cleaning, managing finances, and managing medications.
Paralytic, neuropathic, myopathic, and movement disorders	**Definition:** Paralytic, neuropathic (e.g., paresthesia, numbness, or pain), myopathic (e.g., generalized muscle weakness caused by rhabdomyolysis) or movement disorders (e.g., ataxia) emerging during or after anticancer therapy that substantially affects self-care ADL* for ≥12 months. *E.g., requiring daily assistance beyond what is considered age-appropriate with at least one self-care ADL and/or requiring instrumental aid, such as wheelchair or walking stick, for mobility. **Time of ST:** Date when the condition fulfilling the clinical criteria in the ST definition has persisted for 12 months. **Additional notes:** Examples of self-care ADL include grooming/personal hygiene, dressing, toileting/continence, transferring/ambulating, and eating.
Vocal cord paralysis	**Definition:** Persisting (≥12 months) vocal cord paralysis, either unilateral or bilateral, emerging during or after anticancer therapy, requiring ventilatory support (e.g., non-invasive ventilation) or tracheostomy, or leading to substantially reduced ability or inability to produce speech sounds. **Time of ST:** Date when the condition fulfilling the clinical criteria in the ST definition has persisted for 12 months.
Cytopenia	**Definition:** Profound and permanent cytopenia in one or more hematopoietic cell lines, without evidence of hematopoietic recovery, emerging during or after anticancer therapy and requiring HSCT. **Time of ST:** Date when the patient is referred for HSCT due to cytopenia. **Additional notes:** Myelodysplastic syndromes are captured as second malignant neoplasms.
Immunodeficiency	**Definition:** Permanent immunodeficiency emerging during or after anticancer therapy and requiring HSCT. **Time of ST:** Date when the patient is referred for HSCT due to immunodeficiency. **Additional notes:** Severe leukopenia requiring HSCT is classed as cytopenia.
Second malignant neoplasms and benign central nervous system tumors	**Definition:** Second malignant neoplasms or benign central nervous system tumors emerging during or after anticancer therapy. **Time of ST:** Date when malignant neoplasm or benign central nervous system tumor is diagnosed. **Additional notes:** Non-melanoma skin cancers are not included.

ADL, activities of daily living; HSCT, hematopoietic stem cell transplantation; PEG, percutaneous endoscopic gastrostomy; STFS, severe toxicity-free survival.

^a^ Equating to more than class 3 as per the modified Ross classification system for children aged 0–17.9 years.

^b^ Equating to class 3 or more as per the New York Heart Association Failure Scale for adults.

^c^ Reference (9).

#### 3.1.1. The use of the term “permanent”

##### 3.1.1.1. Description of the problem

One of the generic inclusion criteria of the original ST definitions was that the condition should be anticipated to be *permanent* (or only correctable by an unacceptable treatment). However, valid statistical analyses and thus meaningful comparisons cannot be conducted without adding a *pre-defined timespan* (duration) required for the condition to be classified as an ST, with the timing of the ST defined to be at *the end* of the timespan. When defining this timespan, a compromise was needed between getting the timing of the event as close as possible to the start of the specific health sequelae, and the duration being long enough for the condition to be considered severe irrespective of a potential, future recovery.

##### 3.1.1.2. Solution

Each of the concerned ST definitions was decided to require a duration of minimum 12 months. The length of the timespan was based on two principles: (1) When the condition has persisted for the specified duration, it is likely to be permanent (as specified in the original definition of hepatic failure), and/or (2) when the condition has persisted for the specified duration, it is considered of such severity that it should be included as an ST even though it might improve over time to an extent by which it no longer fulfilled the clinical ST criteria (e.g., heart failure).

#### 3.1.2. Including only conditions occurring *during* treatment for some STs

##### 3.1.2.1. Description of the problem

For several ST definitions, it was required that the conditions occurred during treatment for inclusion as an ST. This was done to increase the likelihood of treatment-related causality. However, causal relation to treatment can never be guaranteed. The selection of certain toxicities with this requirement was based on current knowledge regarding toxicities implying that results would confirm what is already known; but may prevent relevant, new findings.

##### 3.1.2.2. Solution

For all ST definitions, it was decided that all conditions fulfilling the clinical criteria should be included whether they occur during treatment or after treatment.

#### 3.1.3. The use of the term “planned”

##### 3.1.3.1. Description of the problem

For ST definitions including procedures, e.g., surgery, the phrase “*completed or planned*”, referring to the procedure, was used in the original definitions. This, however, needed clarification since it may not always be registered when a procedure is planned, and/or it may be registered inconsequently or with different degrees of delay across physicians/hospitals/countries.

##### 3.1.3.2. Solution

For transplantation (solid organ or bone marrow), it was decided to use the time point when the patient is referred for transplantation (i.e., being put on the relevant transplantation recipient list), which is expected to be consistently registered in the patient's medical record. For other procedures, it was decided that the time point when a procedure is completed is used as the time of the ST. For most procedures, except transplantation, it is expected that there will be no significant time delay between planning and performing a procedure (e.g., implantation of a pacemaker).

#### 3.1.4. Evaluation of activities of daily living (ADL)

##### 3.1.4.1. Description of the problem

ST definitions that include the evaluation of ADL may constitute a challenge when collecting data from medical records: even though it is anticipated that conditions severe enough to substantially affect ADL will in some way be addressed in the patient's medical record, inadequate reporting is a common and limiting factor influencing the evaluation of these conditions. In a prospective setting, the evaluation of ADL is expected to be less of a challenge once the ST criteria are systematically implemented in the outcome evaluations and may further be supported by ADL assessment tools.

Furthermore, ADLs are typically divided into self-care ADL and instrumental ADL, with self-care ADL reflecting the ability to manage basic physical needs such as eating and maintaining personal hygiene, and instrumental ADL including more complex skills, such as the ability to participate in school or manage tasks such as laundry, cooking, etc. (10, 11). Distinguishing between self-care and instrumental ADL was not done consistently in the original definitions. For cognitive dysfunction and psychiatric disease, it was not specified that limitations of ADL should not be a result of physical limitations.

In addition, the original definitions did not specify for how long the condition should affect ADL to be classified as an ST.

Finally, evaluation of ADL for children needs be to age appropriate, since ADL is highly affected by the age of the child (12, 13).

##### 3.1.4.2. Solution

The definitions must consistently specify that “substantially affected ADL” is defined as requiring daily assistance beyond what is considered age appropriate with self-care or instrumental ADL. Concrete examples are now provided with relevant conditions, reserving evaluation of self-care ADL for the physical conditions (e.g., osteonecrosis and neuropathy) and evaluation of instrumental ADL for the cognitive/psychiatric conditions. In preschool children, ADL may be difficult to evaluate but should be based on a level of dependence on others that is not considered age appropriate.

For cognitive dysfunction and psychiatric disease, it was added that the limitations of ADL should not be related to physical limitations.

The concerned consensus definitions were provided with a pre-defined timespan of 12 months at the end of which the condition in question can be classified as an ST.

#### 3.1.5. Lack of standardized evaluation

##### 3.1.5.1. Description of the problem

In contrast to other ST definitions, cognitive dysfunction is not easily identified by a specific diagnosis, unless severe brain damage is present. Objective neuropsychological evaluation and testing are not done systematically or uniformly across treatment centers and should not be a requirement for the ST evaluation. Classification of cognitive dysfunction is dependent on the evaluating physicians who may not weigh the importance and degree of cognitive dysfunction equally. Furthermore, the spectrum describing “normal” cognitive functioning is broad and highly age dependent, particularly when evaluating children and elderly patients (13, 14).

##### 3.1.5.2. Solution

To address the subjectivity and uncertainty accompanying this toxicity, it was decided to grade the outcome into “possible cognitive dysfunction” and “verified cognitive dysfunction”, allowing the analyses to consider a worst-case and a best-case scenario.

#### 3.1.6. Conditions likely to be temporarily affected during cancer therapy

##### 3.1.6.1. Description of the problem

Cognition and psychiatric disorders may be severely but temporarily affected by acute side effects like nausea, pain, and fatigue, present during treatment, and, in some patients, for a prolonged period hereafter.

##### 3.1.6.2. Solution

It was decided to add the requirement that the duration of the conditions included at least 12 months *after the end of cancer therapy* before it could be classified as an ST.

#### 3.1.7. The presence of pre-existing and predisposing conditions that precludes for a specific ST or increased the probability of developing a specific ST

##### 3.1.7.1. Description of the problem

A potential bias is introduced by pre-existing conditions partially fulfilling one or more of the criteria in the ST definition, e.g., unilateral blindness or unilateral deafness that *per se* would increase the probability of complete blindness/deafness, since only one eye/ear needs to be affected during/after treatment to be classified as an ST. A similar problem arises for, e.g., previous acute myocardial infarction or genetic predisposing conditions, e.g., Li-Fraumeni Syndrome, increasing the patient's risk of developing one or more specific STs irrespective of receiving cancer therapy or not.

Another potential bias introduced by the generic criterion, that only conditions occurring during or after cancer therapy will be included, has the consequence that patients with the condition present at the cancer diagnosis, e.g., being blind or deaf, cannot be classified as getting the specific ST.

##### 3.1.7.2. Solution

The generic criterion was extended with the specification that any pre-existing and predisposing conditions must be registered for each ST so these issues can be addressed appropriately in later statistical analyses.

## 4. Discussion

An attempt to apply the previously published physician-defined ST definitions to real-world data revealed problems in the original ST definitions. This paper describes the identified issues and the solutions obtained through a Delphi process and resulting modified consensus definitions of STs.

The most serious problem identified in the original ST definitions was the conditioning on the future by use of the term “permanent”, thereby invalidating the statistical analyses of the occurrence of STs. This issue was resolved by changing the word “permanent” to a pre-defined duration of the clinical criterion in question, at the end of which the condition is classified as an ST. Another issue related to timing concerned the potential bias created by including only conditions occurring during treatment for some of the STs. The remaining changes addressed problems related to potential lack of sufficient information, clarifications of definitions, and challenges for specific STs, all issues that would either lead to problems during the data collection or lead to lack of important knowledge necessary to perform the appropriate statistical analyses.

The modified ST consensus definitions allow for clinically applicable consensus-based reporting and statistical analyses of STs occurring during and after cancer treatment. The clinical applicability is important since evaluation of STs is suggested to be an integrated part of cancer treatment evaluation as a new standard. Even when overall survival and cancer-related event-free survival are similar, toxicity profiles may vary between different protocols because of differences in the use and dose-intensity of steroids, chemotherapy, radiation therapy and hematopoietic stem cell transplantation. Consensus-based capturing of STs will allow for meaningful comparison of toxicity patterns across different treatment protocols.

To be able to perform valid statistical analyses, any potentially reversible and fluctuating condition requires a pre-defined duration at the end of which it can be classified as an ST. Examples of such conditions are neuropathy and heart failure. Including this pre-defined duration in the definition has the additional consequence that a patient who develops, e.g., severe heart failure and dies before the end of the required timespan will not be counted as having had the ST, hence potentially underestimating the occurrence of clinically relevant severe conditions if the length of the required timespan is too long. The magnitude of this problem could be explored by considering different lengths of the timespan for the different conditions.

For all the definitions, a general consideration included determining whether a condition is truly related to the cancer therapy. Certain toxicities are well established as related to a specific treatment, e.g., vincristine-induced neuropathy, but others are more difficult to link to the cancer treatment with certainty, especially if the toxicity also occur with a relatively high frequency in the background population, and in general, causality cannot be guaranteed. For example, amputation as a result of a car accident might be tempting to exclude as an event, since it was most likely caused by another etiology than cancer, but impaired concentration caused by cancer treatment could also have been a contributing factor. However, such sorting cannot be done systematically and would introduce bias, since the sorting would depend on unverifiable assumptions and in many cases reflect pre-existing knowledge about toxicities, thereby delay or even suppress new findings. Hence, it is necessary to include all cases that fulfill the clinical criteria in the ST definition, which mirrors the approach in survival analyses where death of any cause also is included. The same arguments have the consequence that all conditions fulfilling the clinical criteria should be included whether they occur during treatment or after treatment. A potential overestimation caused by including conditions occurring after treatment in the ST definitions may be addressed by robustness analyses that include only conditions occurring during treatment for conditions likely to occur for non-cancer-related reasons, e.g., heart disease among elderly survivors. For childhood cancers this is considered less a challenge since the severe conditions included in the ST definitions very rarely would occur in children without cancer.

Pre-existing conditions may lead to increased or decreased risk of the occurrence of an ST irrespective of receiving cancer therapy or not. Excluding all patients with any of these conditions from the start may be a problem for composite outcomes like STFS or the burden of multiple toxicities since this would lead to a selected population. On the other hand, including the patient that can never be classified as having a specific toxicity will introduce bias towards a lower burden of STs and/or potential confounding of comparisons of treatment protocols. How to address these conditions appropriately in later statistical analyses will depend on the actual circumstances. The frequency of pre-existing conditions relevant for the ST measure (e.g., co-morbidities like diabetes or cognitive dysfunction) have not been evaluated in ALL cohorts previously but will be a relevant outcome in the planned international evaluation of STs across five childhood ALL cohorts.

Several ST definitions include a costly procedure, e.g., implantation of a pacemaker, which may lead to confounding by socioeconomic factors and/or availability of free health care services (15–17). Also, variation in clinical practice (e.g., surgery for osteonecrosis) and cultural differences (e.g., in the evaluation of ADL) could affect rates of the individual STs and, therefore, also the combined measures like STFS or the burden of multiple toxicities.

Describing the occurrence of toxicities is often based on time to first event as done in the STFS approach, which is highly relevant, but does not describe the burden of multiple toxicities that an individual survivor may suffer from. The total burden of toxicities involves multiple types of events as well as recurrent events. A cumulative burden can be estimated using the mean cumulative count method, estimating the expected number of events of interest as a function of time (18). The events of interest must be chosen with care to reflect which aspect of the total burden is in focus for the specific analysis: which types of events to include as well as which types of events to count repeatedly will depend on the focus of the analysis. A focus on the burden on the specialized health care system will lead to a different choice than a focus on the burden on the patient's everyday life. It should be noted that each event will be weighted equally in the analysis, which must be considered when deciding which types of events to include and which types of events may be counted repeatedly since this has major consequence for the interpretation of the results. Therefore, the definition of the burden should be considered carefully.

For the STs presented here, one approach would be to look at the number of *different* toxicities, i.e., counting each type of ST only once in the same patient, while different types of STs in the same patient would count as separate events. This would provide a more comprehensive estimate compared to only evaluating the time to first ST but would still potentially underestimate the burden on the patient as well as on the specialized health care system since several types of STs may occur more than once, e.g., osteonecrosis in several joints and second malignant cancers. These recurring events would be “ignored” with the beforementioned approach, despite adding to the disease burden for the individual survivor as well as for the specialized health care system.

Although relevant for evaluation of the objectively most serious consequences of being treated for a cancer, the STs presented here are not sufficient for an evaluation of the total burden of therapy from the perspective of the patients who do not measure their quality of life in numbers of toxicities. Of note, the overall quality of life for the individual survivor reflects a complex combination of physiological, psychosocial, and socioeconomic factors (19, 20), not encompassed by the evaluation of the selected STs, which should rather be considered a first step towards integration of treatment sequelae in the evaluation of cancer treatment. The most severe toxicities only constitute the tip of the iceberg, and the total burden of therapy includes also lower-grade and subjective conditions, which may be equally burdensome for the individual survivor. However, capturing of the objectively most severe toxicities is an important step towards evaluating overall quality of life for cancer survivors and will allow for subsequent inclusion of the perspective from patients living with one or more STs.

The current modified ST consensus definitions reflect an inherent need to make the original ST definitions clinically applicable, but these revised definitions do not constitute a definitive or perfect measure for the evaluation of severe long-term toxicities. In parallel with changes in treatment strategies and addition of novel treatment agents, other toxicities may be relevant to include, or additional modifications of the current ST definitions may be considered relevant. The presented STs are intended for general use in cancer treatment evaluation in children and adults, but the level of severity of a toxicity to be considered an unacceptable trade-off for cure may depend on the context. Different cut-points may be needed for elderly patients compared to childhood cancer survivors who may live many years following cancer. Likewise, a higher burden of toxicities must be accepted if needed to achieve disease control (being the primary treatment goal) for cancers with low survival probabilities. Other special considerations and adaptions may be relevant for specific cancer types, e.g., amputation as part of the curative treatment in a patient with osteosarcoma should not be considered an ST.

Evaluation of severe toxicities should be integrated in cancer treatment evaluation alongside traditional treatment outcomes. The strength of the concept of consensus-based STs lies in the global decision to capture and report severe toxicities as a routine part of treatment evaluation rather than in the consensus definitions themselves. Consensus-based capturing of STs will allow for meaningful comparison of toxicity patterns across different treatment protocols and different cohorts, thereby paving the way for future research on risk factors, potentially facilitating modifications in cancer therapy and early targeted interventions with the overall goal to further reduce toxicities without compromising cure.

## Data Availability

The original contributions presented in the study are included in the article/**Supplementary Material**, further inquiries can be directed to the corresponding author.
