# Supplementary material for: Physician-defined severe toxicities occurring during and after cancer treatment: Modified consensus definitions and clinical applicability in the evaluation of cancer treatment
Source: Front Pediatr. 2023 Apr 26;11:1155449. doi: 10.3389/fped.2023.1155449 (PMC10171426; doi:10.3389/fped.2023.1155449)
Supplement: Supplementary file 1 [file Datasheet1.docx]

Supplementary Material

Physician-Defined Severe Toxicities Occurring During and After Cancer Treatment: Modified Consensus Definitions and Clinical Applicability in the Evaluation of Cancer Treatment

Camilla Grud Nielsen, Birthe Lykke Thomsen, Bodil Als-Nielsen, Rachel Conyers, Sima Jeha, Marion Mateos, Wojciech Mlynarski, Rob Pieters, Mathias Rathe, Kjeld Schmiegelow, Liv Andrés-Jensen*, on behalf of the Ponte di Legno Severe Toxicity Working Group

*** Correspondence:**

Liv Andrés-Jensen
[liv.andres-jensen.02@regionh.dk](mailto:liv.andres-jensen.02@regionh.dk)

Table of content

[1 Ponte di Legno Severe Toxicity Working Group 3](#_Toc126061305)

[2 Delphi process 4](#_Toc126061306)

[2.1 Methods 4](#_Toc126061307)

[2.2 Results 7](#_Toc126061308)

[2.2.1 Quantitative results 8](#_Toc126061309)

[2.2.2 Qualitative results 9](#_Toc126061310)

[3 Original and modified ST definitions 14](#_Toc126061311)

[3.1 Hearing loss 14](#_Toc126061312)

[3.2 Blindness 15](#_Toc126061313)

[3.3 Heart failure 16](#_Toc126061314)

[3.4 Coronary artery disease 19](#_Toc126061315)

[3.5 Arrhythmia 20](#_Toc126061316)

[3.6 Heart valve disease 21](#_Toc126061317)

[3.7 Gastrointestinal failure 22](#_Toc126061318)

[3.8 Hepatic failure 24](#_Toc126061319)

[3.9 Insulin dependent diabetes 26](#_Toc126061320)

[3.10 Renal failure 27](#_Toc126061321)

[3.11 Pulmonary failure 28](#_Toc126061322)

[3.12 Osteonecrosis 29](#_Toc126061323)

[3.13 Amputation and physical deformation 31](#_Toc126061324)

[3.14 Cognitive dysfunction 34](#_Toc126061325)

[3.15 Seizures 37](#_Toc126061326)

[3.16 Psychiatric disease 39](#_Toc126061327)

[3.17 Paralytic, myopathic and movement disorders 41](#_Toc126061328)

[3.18 Vocal cord paralysis 43](#_Toc126061329)

[3.19 Cytopenia 44](#_Toc126061330)

[3.20 Immunodeficiency 45](#_Toc126061331)

[3.21 Second malignant neoplasms and benign central nervous system tumors 46](#_Toc126061332)

[4 Original and modified generic ST inclusion criteria 47](#_Toc126061333)

[5 Reference list 51](#_Toc126061334)

# Ponte di Legno Severe Toxicity Working Group

The large international collaboration named the Ponte di Legno Working Group (PdL) represents 17 childhood acute lymphoblastic leukemia (ALL) study groups and institutions worldwide (1). In 2019 the PdL group initiated a project addressing severe, treatment-related toxicities. The aim of the project was to prioritize and define the objectively most severe toxicities for the future reporting of severe toxicity-free survival (STFS) alongside traditional treatment outcomes. This work by the Ponte di Legno Severe Toxicity Working Group (PTWG) resulted in consensus definitions of 21 Severe Toxicities (2), and the current paper is based on this work.

# Delphi process

## Methods

The Delphi process is a structured process that uses series of questionnaires or “rounds” to gather both quantitative and qualitative information on the level of agreement on a subject (3). The modified Delphi process combines the use of anonymous questionnaires and plenary meetings of the panelists to discuss the reasons for potential disagreements and the need for clarifications. Furthermore, all panelists receive both quantitative and qualitative feedback between rounds, allowing one to reconsider previous opinions when considering the comments from other panelists. One major advantage of the Delphi process is that panelists participate and comment anonymously avoiding the consensus process being dominated by one or a few experts.

Aim: The aim of this Delphi process was to establish consensus on the modified ST definitions.

Panelists: The panelists included 10 experts within the field of childhood ALL and/or toxicity, in addition to one biostatistician with expertise in time-to-event analyses (Table 1.1).

Indicators: The starting point was the previously published consensus definitions of 21 STs (2). Based on plenary discussions between the steering group (Table 1.2) and principal investigators of the planned international evaluation of STs (Table 1.3), the steering group proposed 21 modified ST definitions that served as indicators in the Delphi process.

Selection criteria: The 21 STs were initially selected and defined based on five generic inclusion criteria:

1. Not present prior to ALL diagnosis
2. Symptomatic
3. Objective
4. Unacceptable severity
5. Permanent or only correctable by unacceptable treatment

Delphi process: Panelists were asked to rate each definition by stating their level of agreement as either high, medium, or low. If panelists indicated medium or low level of agreement, they would be required to leave a comment with suggestions for improvement of the definition in question, or at least to elaborate on the reason for the disagreement.

Feedback: Between rounds panelists received anonymized quantitative and qualitative (anonymously) results from the previous round. Plenary meetings were held allowing for discussions on potential disagreements. Before each new round, suggestions for improvements were circulated from the steering group (details available below) based on feedback from the previous round and plenary discussions.

Consensus: Consensus was defined a priori as requiring 100% consensus, i.e., high level of agreement for all definitions from all panelist without a defined number of rounds to reach this (4).

**Table 1.1 Delphi process panelists**

| **Australia** | Marion K Mateos^1,2,3^ Rachel Conyers^4,5,6^ |
| --- | --- |
| **DCOG** | Rob Pieters^7^ |
| **NOPHO** | Bodil Als-Nielsen^8^ Birthe Lykke Thomsen, biostatistician^8^ Camilla Grud Nielsen^8^ Kjeld Schmiegelow^8^ Liv Andrés-Jensen^8^ Mathias Rathe^9,10^ |
| **Poland** | Wojciech Mlynarski^11^ |
| **SJCRH** | Sima Jeha^12^ |

DCOG=Dutch Childhood Oncology Group, NOPHO=Nordic Society of Paediatric Haematology and Oncology, SJCRH=St Jude Children’s Research Hospital

A**ffiliations**

1. Kids Cancer Centre, Sydney Children’s Hospital Randwick, Sydney, Australia
2. Discipline of Paediatrics and Child Health, School of Clinical Medicine, UNSW Medicine & Health, UNSW Sydney
3. Children’s Cancer Institute, Lowy Cancer Research Centre, UNSW, Sydney, Australia
4. Department of Paediatrics, University of Melbourne, Melbourne, Australia
5. Pharmacogenomics, Stem Cell Biology, Murdoch Children’s Research Institute, Melbourne, Australia
6. Children’s Cancer Centre, The Royal Children’s Hospital, Melbourne, Australia
7. Princess Maxima Center for Pediatric Oncology, Utrecht, The Netherlands
8. Department of Pediatrics and Adolescent Medicine, Copenhagen University Hospital, Denmark
9. Hans Christian Andersen’s Children’s Hospital, Odense University Hospital, Denmark
10. Department of Clinical Research, University of Southern Denmark, Odense, Denmark
11. Department of Pediatrics, Oncology & Hematology, Medical University of Lodz, Poland
12. Department of Oncology, St Jude Children’s Research Hospital, Memphis, TN, USA

**Table 1.2 Steering group**

| NOPHO | Bodil Als-Nielsen  Birthe Lykke Thomsen, biostatistician  Camilla Grud Nielsen  Kjeld Schmiegelow  Liv Andrés-Jensen  Mathias Rathe |
| --- | --- |

**Table 1.3 Principal investigators of the planned international evaluation of STs**

| Australia | Marion K Mateos  Rachel Conyers |
| --- | --- |
| DCOG | Rob Pieters |
| NOPHO | Kjeld Schmiegelow |
| Poland | Wojciech Mlynarski |
| SJCRH | Sima Jeha |

## Results

Three Delphi rounds were performed before reaching full consensus for all indicators (see section 1). The response rate was 100% in all rounds. Panelists participating in the first round included 10 ALL and/or toxicity experts. The biostatistician was also part of round 2 and round 3. An overview of quantitative and qualitative results is presented below.

### Quantitative results

| **Quantitative results – Delphi process** | | | | | | | | | |
| --- | --- | --- | --- | --- | --- | --- | --- | --- | --- |
| **Severe Toxicity (indicator)** | **Round 1** | | | **Round 2** | | | **Round 3** | | |
|  | **H** | **M** | **L** | **H** | **M** | **L** | **H** | **M** | **L** |
| Hearing loss | 100 | 0 | 0 | 91 | 9 | 0 | 100 | 0 | 0 |
| Blindness | 100 | 0 | 0 | 100 | 0 | 0 | 100 | 0 | 0 |
| Heart failure | 100 | 0 | 0 | 91 | 9 | 0 | 100 | 0 | 0 |
| Coronary artery disease | 100 | 0 | 0 | 100 | 0 | 0 | 100 | 0 | 0 |
| Arrhythmia | 90 | 0 | 10 | 100 | 0 | 0 | 100 | 0 | 0 |
| Heart valve dysfunction | 100 | 0 | 0 | 100 | 0 | 0 | 100 | 0 | 0 |
| Gastrointestinal failure | 80 | 20 | 0 | 82 | 18 | 0 | 100 | 0 | 0 |
| Hepatic failure | 80 | 10 | 10 | 91 | 9 | 0 | 100 | 0 | 0 |
| Insulin dependent diabetes | 90 | 10 | 0 | 91 | 9 | 0 | 100 | 0 | 0 |
| Renal failure | 80 | 20 | 0 | 91 | 9 | 0 | 100 | 0 | 0 |
| Pulmonary failure | 90 | 10 | 0 | 100 | 0 | 0 | 100 | 0 | 0 |
| Osteonecrosis | 90 | 10 | 0 | 100 | 0 | 0 | 100 | 0 | 0 |
| Amputation and physical deformation | 100 | 0 | 0 | 91 | 9 | 0 | 100 | 0 | 0 |
| Cognitive dysfunction | 60 | 40 | 0 | 91 | 9 | 0 | 100 | 0 | 0 |
| Seizures | 70 | 20 | 10 | 91 | 9 | 0 | 100 | 0 | 0 |
| Psychiatric disease | 80 | 10 | 10 | 100 | 0 | 0 | 100 | 0 | 0 |
| Paralytic, neuropathic, myopathic, and movement disorders | 100 | 0 | 0 | 100 | 0 | 0 | 100 | 0 | 0 |
| Vocal cord paralysis | 90 | 0 | 10 | 91 | 9 | 0 | 100 | 0 | 0 |
| Cytopenia | 90 | 10 | 0 | 100 | 0 | 0 | 100 | 0 | 0 |
| Immunodeficiency | 100 | 0 | 0 | 100 | 0 | 0 | 100 | 0 | 0 |
| Second malignant neoplasms and benign CNS tumors | 90 | 10 | 0 | 91 | 9 | 0 | 100 | 0 | 0 |

Numbers indicate the percentage of panelists. Level of agreement: H=high, M=medium, L=low

### Qualitative results

The qualitative results include feedback from the panelists on the modified ST definitions as well some general comments received between rounds. The feedback is presented in themes, i.e., comments regarding the same issue are only described once. Comments addressing spelling and phrasing are not shown. General comments are shown separately. All comments in their full length and exact wording are available upon request.

| **Qualitative results – Delphi process** | | | |
| --- | --- | --- | --- |
| **Severe Toxicity (indicator)** | **Round 1** | **Round 2** | **Round 3** |
| Hearing loss | -Suggestion to include conditions emerging after ending anticancer therapy | -Discussion if it should be required that some degree of hearing loss is detected during anticancer therapy.  -Suggestion to change the word “permanent” to “persisting” | - |
| Blindness | - | - | -Discussion regarding including conditions emerging during and after anticancer therapy |
| Heart failure | - | -Suggestion to change the word “permanent” to “persisting” | - |
| Coronary artery disease | - | - | - |
| Arrhythmia | - | - | - |
| Heart valve dysfunction | - | - | - |
| Gastrointestinal failure | -Suggestion to include conditions emerging after ending anticancer therapy if considered associated with a treatment-related toxicity, e.g., as part of cGVHD following HSCT. | -It was discussed that the inclusion of different conditions within the same ST must rely on the same criteria and that in general, inclusion of conditions must not depend on what kind of treatment the patient has received, since this would lead to bias.  -Suggestion to elaborate in the note that only PEG-tube inserted for physical reasons are included.  -Suggestion to change the word “permanent” to “persisting” | - |
| Hepatic failure | -Suggestion to make the definition clearer and tighter, for example by moving part of the definition describing typical symptoms to the ‘Additional notes’ section. | -Suggestion to change the word “permanent” to “persisting” | - |
| Insulin dependent diabetes | - | -Suggestion to change the word “permanent” to “persisting” | -Discussion regarding including conditions emerging during and after anticancer therapy |
| Renal failure | - | -Suggestion to change the word “permanent” to “persisting” | -Discussion regarding including conditions emerging during and after anticancer therapy |
| Pulmonary failure | - | - | - |
| Osteonecrosis | - | - | - |
| Amputation and physical deformation | -Suggestion to include conditions emerging after anticancer therapy if associated with a treatment-related toxicity, e.g., scleroderma following chronic GVHD. | -It was discussed that the inclusion of different conditions within the same ST must rely on the same criteria and that in general, inclusion of conditions must not depend on what kind of treatment the patient has received, since this would lead to bias. | - |
| Cognitive dysfunction | -It was found difficult to distinguish “treatment-related” cases from “normal cognitive issues of childhood”, e.g., ADHD, ASD.  -Suggestion to mention that although neuropsychological testing is likely for patients with severe neurocognitive dysfunction, such testing is not required to provide the STFS scoring  -It was discussed if certain criteria should be met for the evaluation of this condition, due to the lack of standardized evaluation across different study groups.  -Suggestion to expand the duration criteria to be 12 months after ending anticancer therapy” since it is well-known that many patients can be very affected cognitively during also maintenance therapy and then may improve after ending therapy. | -Suggestion to delete specific criteria that should be met for evaluation of this condition, since it would lead to bias if patients were excluded from the start. Suggestion to specify in ‘Additional notes’ that relevant pre-existing conditions for this ST include not having met normal developmental milestones or evidence of developmental delay at time of diagnosis. | - |
| Seizures | - It was discussed if the definition should be expanded to require having >1 seizure per year in addition to be classified as having drug-resistant epilepsy. Argues against this suggestion included that this would be our subjective opinion, and the most appropriate would be to align with the ILAE definition (as originally done) | -Suggestion to change the wording slightly making the definition a direct quote of the ILAE definition. | -Suggestion to change the definition to include seizures emerging both during and after anticancer therapy |
| Psychiatric disease | -It was discussed that a significant proportion of the background population ask for psychological support which should not be enough for classification as an ST. The definition states, however, that the condition should affect ADL substantially and persist for ≥12 months after ending anticancer therapy, to be classified as an ST.  - It was discussed how to handle patients with known psychiatric disease prior to cancer diagnosis, which could be a significant proportion. Suggestion to register known relevant pre-existing conditions corresponding to any ST to allow the analyses to take such conditions into account. |  | -Discussion regarding including conditions emerging during and after anticancer therapy |
| Paralytic, neuropathic, myopathic, and movement disorders | - | - | -Discussion regarding including conditions emerging during and after anticancer therapy |
| Vocal cord paralysis | -Suggestion to specify that tracheostomy doesn’t necessarily mean ventilatory support is required and to add an example of ventilatory support which does not include tracheostomy. | -Suggestion to change the word “permanent” to “persisting” | -Discussion regarding including conditions emerging during and after anticancer therapy |
| Cytopenia | -Suggestion to define cytopenia with specific blood counts numbers.  -Suggestion to define possible underlying conditions to guide the data collector. | - | -Discussion regarding including conditions emerging during and after anticancer therapy |
| Immunodeficiency | -Suggestion to register known underlying conditions for all STs | - | -Discussion regarding including conditions emerging during and after anticancer therapy |
| Second malignant neoplasms and benign CNS tumours | - | -It was questioned why only melanoma skin cancer is included. | - |

| **General comments** | | |
| --- | --- | --- |
| **Round 1** | **Round 2** | **Round 3** |
| -Discussed how to specify the timing of each of ST (time of event). | -Suggestion to change the word “permanent” to “persisting” (except for cytopenia and immunodeficiency where the criteria depends on requiring HSCT). | - Discussion regarding including all conditions fulfilling the clinical criteria in the ST definition whether occurring during or after cancer treatment |
| -Suggestion to register relevant, known pre-existing and predisposing conditions corresponding any STs to allow the statistical analyses to take these conditions into account, since they may affect an individual’s risk of developing certain STs. | -Discussion if there should be an upper limit for how long after end of treatment the occurrence of specific STs (that may emerge after end of therapy) can be classified as an ST. It was discussed that treatment-related causality in general cannot be guaranteed, but it is acknowledged that causality will be less certain after many years of follow-up. |  |
| -Suggestion to define ADL as age-appropriate |  |  |
| -Suggestion to specify the meaning of the 12 months criteria. |  |  |

# Original and modified ST definitions

Original and modified ST definitions are shown, as well as the underlying considerations.

## Hearing loss

| **Original ST definition** | **Additional notes** |
| --- | --- |
| Permanent bilateral hearing loss emerging during anticancer therapy and defined as need for cochlear implant (completed or planned), or >40 dB hearing loss at ≤2 kHz | - |

| **Modified ST definition** | **Additional notes** | **Time of ST** |
| --- | --- | --- |
| Persisting bilateral hearing loss emerging during or after anticancer therapy and defined as >40 dB hearing loss at ≤2 kHz that persists for ≥12 months or requiring cochlear implant. | - | Date when at least one of the criteria are met for both ears, i.e., both ears having audiometry identified >40 dB hearing loss at ≤2 kHz that has persisted for ≥12 months and/or cochlear implant surgery (whichever occurs first). |

Changes and considerations:

The word “permanent” is replaced with “persisting”, and a pre-defined timespan of 12 months was necessary to add to the word “*persisting*” based on generic considerations regarding determining when a condition is to be classified as an ST. Conditions fulfilling the clinical criteria are included whether they occur during treatment or after treatment. This decision was based on general considerations regarding treatment-related causality and the potential bias introduced by only including conditions emerging during cancer treatment. Furthermore, literature supports that ototoxicity following treatment with platinum compounds and radiation therapy may occur both during and after cancer therapy and may also be progressive (5–8).

The phrase “completed or planned” is deleted from the original definition based on the general considerations regarding procedures.

## Blindness

| **Original ST definition** | **Additional notes** |
| --- | --- |
| Untreatable blindness emerging during anticancer therapy, defined as visual acuity of <20/200 or a corresponding visual field loss to <10° in the stronger eye with the best possible correction. | - |

| **Modified ST definition** | **Additional notes** | **Time of ST** |
| --- | --- | --- |
| Untreatable blindness emerging during or after anticancer therapy, defined as visual acuity of <20/200 or a corresponding visual field loss to <10° in the stronger eye with the best possible correction. | - | Date when blindness is identified as visual acuity of <20/200 or a corresponding visual field loss <10° in the stronger eye with the best possible correction. |

Changes and considerations:

Conditions fulfilling the clinical criteria are included whether they occur during treatment or after treatment. This decision was based on general considerations regarding treatment-related causality and the potential bias introduced by only including conditions emerging during cancer treatment.

It is expected that a patient with such severe visual impairment will have undergone an eyesight test.

## Heart failure

| **Original ST definition** | **Additional notes** |
| --- | --- |
| Permanent symptomatic cardiac dysfunction emerging during or after anticancer therapy and defined by a decrease in left ventricular ejection fraction to a value <40% or fractional shortening to <20% and one of the following:   - Age 0-1 years: marked tachypnoea or diaphoresis with feeding or prolonged feeding times with growth failure or tachypnoea, retractions, grunting, or diaphoresis at rest.* - Age 1-17.9 years: marked dyspnoea on exertion or at rest.* - Age ≥ 18 years: marked dyspnoea, palpitations or anginal pain on exertion or at rest.**   *Equating to more than class 3 as per the modified Ross Classification System for children aged 0-17.9 years **Equating to class 3 or more as per the New York Heart Association Failure Scale for adults | Screening of patients with echocardiographic measures are generally not required for inclusion as a severe toxicity, but echocardiographic measures are included in this definition because we expect that all patients with symptoms will be identified and have one done. Echocardiographic measures are provided because international surveillance guidelines accept its use as the primary surveillance tool for cardiotoxicity. It is expected that a repeat echocardiograms will be done at least 1 week apart to confirm cardiac dysfunction. |

| **Modified ST definition** | **Additional notes** | **Time of ST** |
| --- | --- | --- |
| Persisting (≥12 months), symptomatic cardiac dysfunction emerging during or after anticancer therapy and defined by a decrease in left ventricular ejection fraction to a value <40% or fractional shortening to <20% and one of the following:   - Age 0-1 years: marked tachypnoea or diaphoresis with feeding or prolonged feeding times with growth failure or tachypnoea, retractions, grunting, or diaphoresis at rest* - Age 1-17.9 years: marked dyspnea on exertion or at rest* - Age ≥18 years: marked dyspnea, palpitations or anginal pain on exertion or at rest**   OR   - Symptomatic cardiac dysfunction requiring heart transplantation   *Equating to more than class 3 as per the modified Ross Classification System for children aged 0-17.9 years **Equating to class 3 or more as per the New York Heart Association Failure Scale for adults | Screening of patients with echocardiographic measures is not required for inclusion as a severe toxicity, but echocardiographic measures are required. Echocardiographic measures are provided because international surveillance guidelines accept its use as the primary surveillance tool for cardiotoxicity. It is expected that a repeat echocardiogram will be done at least 1 week apart to confirm cardiac dysfunction. | Date when the condition fulfilling the clinical and paraclinical criteria has persisted for 12 months, or when the patient is referred for transplantation (whichever occurs first). |

Changes and considerations:

Heart failure is mainly associated with irradiation to the chest and high cumulative doses of anthracyclines (10,11). However, even lower cumulative doses of anthracyclines can lead to asymptomatic echocardiographic changes that may progress over time resulting in symptomatic left ventricular dysfunction (12).

The word “permanent” is replaced with “persisting”, and a pre-defined timespan of 12 months was necessary to add to the word “*persisting*” based on generic considerations regarding determining when a condition is to be classified as an ST. It cannot be stated with certainty that symptomatic heart failure is irreversible after 12 months, however, it will at that timepoint be considered of such severity that it should be included as a Severe Toxicity despite such uncertainty. Also, it has been shown that even if heart failure improves, it is usually followed by a second decline in function (13).

Children with LVEF <40% are expected to receive medical treatment. The criteria in the ST definition are only fulfilled if the patient is symptomatic and is not related to whether the patient receives medical treatment for the heart failure or not (since the treatment is not considered unacceptable).

Cardiotoxic treatment may cause diastolic dysfunction which is also described in patients with preserved LVEF (14). However, at current time echocardiographic measures of diastolic dysfunction are not used systematically, why LVEF and fractional shortening are applied for this definition as the most appropriate measure (11).

The other ST definitions that include organ failure, e.g., renal failure and hepatic failure, also include organ transplantation as an outcome. In alignment with that, heart transplantation is added as an outcome for this definition.

## Coronary artery disease

| **Original ST definition** | **Additional notes** |
| --- | --- |
| Coronary artery disease emerging during or after anticancer therapy and resulting in   - myocardial infarction, or - requiring angioplasty (balloon or stent), or - coronary bypass surgery (completed or planned) | - |

| **Modified ST definition** | **Additional notes** | **Time of ST** |
| --- | --- | --- |
| Coronary artery disease emerging during or after anticancer therapy and resulting in   - myocardial infarction, or - requiring angioplasty (balloon or stent), or - coronary bypass surgery. | - | Date of myocardial infarction or when angioplasty (balloon or stent) or coronary bypass surgery is performed (whichever occurs first). |

Changes and considerations:

The phrase “*completed or planned”* is deleted from the original definition based on the general considerations regarding procedures.

## Arrhythmia

| **Original ST definition** | **Additional notes** |
| --- | --- |
| Arrhythmia emerging during or after anticancer therapy, requiring a pacemaker or an implantable cardioverter defibrillator (completed or planned). | Known underlying predisposing condition likely to explain the arrythmia is reported at time of severe toxicity data capture. |

| **Modified ST definition** | **Additional notes** | **Time of ST** |
| --- | --- | --- |
| Arrhythmia emerging during or after anticancer therapy, requiring a pacemaker or an implantable cardioverter defibrillator. | - | Date when pacemaker or ICD is implanted. |

Changes and considerations:

The phrase “*completed or planned”* is deleted from the original definition based on generic considerations regarding procedures.

The note describing underlying conditions is deleted based on general considerations regarding pre-existing conditions.

## Heart valve disease

| **Original ST definition** | **Additional notes** |
| --- | --- |
| Heart valve dysfunction emerging during or after anticancer therapy and requiring surgical valve replacement (completed or planned). | - |

| **Modified ST definition** | **Additional notes** | **Time of ST** |
| --- | --- | --- |
| Heart valve dysfunction emerging during or after anticancer therapy and requiring surgical valve replacement. | - | Date when surgical valve replacement is performed. |

Changes and considerations:

The phrase “*completed or planned*” is deleted from the original definition based on general considerations regarding procedures.

## Gastrointestinal failure

| **Original ST definition** | **Additional notes** |
| --- | --- |
| Gastrointestinal failure emerging during anticancer therapy, resulting in   - permanent (at time of evaluation) need for parenteral nutrition, or - placement of a permanent PEG tube due to physical inability to eat or swallow, or - placement of permanent stoma (completed or planned). | Underlying conditions include critical reduction in gastrointestinal tract mass and all other conditions leading to the described gastrointestinal failure |

| **Modified ST definition** | **Additional notes** | **Time of ST** |
| --- | --- | --- |
| Gastrointestinal failure emerging during or after anticancer therapy, resulting in   - persistently (≥12 months) requiring parenteral nutrition, or - placement of a PEG tube due to physical inability to eat or swallow that persists for ≥12 months, or - placement of a stoma that persists for ≥12 months. | - | Date when the condition fulfilling the clinical criteria in the ST definition has persisted for 12 months. |

Changes and consideration:

The word “permanent” is replaced with “persisting”, and a pre-defined timespan of 12 months was necessary to add to the word “*persisting*” based on generic considerations regarding determining when a condition is to be classified as an ST. It is acknowledged that having a PEG-tube, stoma, or parenteral nutrition for 12 months, doesn’t necessarily indicate that it will be permanent. There can be various reasons for the prolonged placement of a PEG-tube (including psychological conditions), however, the need for PEG-tube, stoma, or parenteral nutrition for ≥12 months is considered of such severity that it should be included as a Severe Toxicity regardless.

Conditions fulfilling the clinical criteria are included whether they occur during treatment or after treatment. This decision was based on general considerations regarding treatment-related causality and the potential bias introduced by only including conditions emerging during cancer treatment.

In the original definition the following phrase *“gastrointestinal failure resulting in permanent (at time of evaluation) need for parenteral nutrition”* is used. The phrase *“at time of evaluation”* is deleted from the original definition since results should not depend on the timepoint of data capture, which would challenge the possibility to compare a retrospective study with future prospective studies.

The note mentioning underlying conditions is deleted. Known predisposing conditions will be captured for all patients and all Severe Toxicities.

## Hepatic failure

| **Original ST definition** | **Additional notes** |
| --- | --- |
| Severe and permanent hepatobiliary failure emerging during or after anticancer therapy, and defined as any of the following:   - Symptomatic~~*~~, decompensated liver disease including cirrhosis and portal hypertension that is not responsive to pharmacologic and endoscopic management** and is persisting for ≥ 12 months;   OR   - Any hepatobiliary failure requiring liver transplantation (completed or planned)   *Typical symptoms include fatigue, gum bleeding, epistaxis, itching, and icterus in all age groups in addition to impaired growth and delayed puberty in children.  ** Patients reaching resolution after ligation and sclerotherapy for varices are excluded, patients receiving a shunt are included because shunts are intended for refractory disease, most often as a bridge to liver transplant. | - |

| **Modified ST definition** | **Additional notes** | **Time of ST** |
| --- | --- | --- |
| Severe and persistent (≥12 months) hepatobiliary failure emerging during or after anticancer therapy, and defined as any of the following:   - Symptomatic, decompensated liver disease including cirrhosis and portal hypertension that is not responsive to pharmacologic and endoscopic management**   OR   - Any hepatobiliary failure requiring liver transplantation. | Typical symptoms of hepatic failure include fatigue, gum bleeding, epistaxis, itching, and icterus in all age groups in addition to impaired growth and delayed puberty in children.  Patients who undergo a portosystemic shunt for hepatic disease are included in this definition because shunts are usually reserved for refractory disease, which may serve as a bridge to liver transplant. | Date when the condition fulfilling the clinical criteria in the ST definition has persisted for 12 months or when the patient is approved for transplantation (whichever occurs first). |

Changes and consideration:

The phrase *“completed or planned”* is deleted from the original definition based on general considerations regarding procedures.

The phrase *“Patients reaching resolution after ligation and sclerotherapy for varices are excluded”* is deleted from the original definition, since it is redundant.

The word “permanent” is replaced with “persisting” based on general discussions regarding the use of the word “permanent”. A pre-defined timespan of 12 months was already included in this definition.

Examples of symptoms and elaboration on how to approach patients receiving a portosystemic shunt are moved to the ‘Additional notes’ for clarification.

## Insulin dependent diabetes

| **Original ST definition** | **Additional notes** |
| --- | --- |
| Permanent insulin dependent diabetes emerging during anticancer therapy | Insulin dependent diabetes is treatable; however, is included because of substantial risk of cardiovascular disease and end-organ failure |

| **Modified ST definition** | **Additional notes** | **Time of ST** |
| --- | --- | --- |
| Persisting (≥12 months) insulin dependent diabetes emerging during or after anticancer therapy. | Insulin dependent diabetes is treatable; however, is included because of substantial risk of cardiovascular disease and end-organ failure. | Date when the condition fulfilling the clinical criteria in the ST definition has persisted for 12 months. |

Changes and considerations:

The word “permanent” is replaced with “persisting”, and a pre-defined timespan of 12 months is added to the word *“persisting”* based on general considerations regarding determining when a condition is to be classified as a Severe Toxicity. Patients with persisting need for insulin after 12 months are likely to continue to require insulin (15).

Conditions fulfilling the clinical criteria are included whether they occur during treatment or after treatment. This decision was based on general considerations regarding treatment-related causality and the potential bias introduced by only including conditions emerging during cancer treatment.

## Renal failure

| **Original ST definition** | **Additional notes** |
| --- | --- |
| Permanent loss of kidney function emerging during anticancer therapy that requires dialyses or renal transplantation (completed or planned) | - |

| **Modified ST definition** | **Additional notes** | **Time of ST** |
| --- | --- | --- |
| Persisting (≥12 months) loss of kidney function emerging during or after anticancer therapy that requires dialysis or renal transplantation. | - | Date when the condition fulfilling the clinical criteria in the ST definition has persisted for 12 months or when the patient is approved for transplantation (whichever occurs first). |

Changes and considerations:

The phrase *“completed or planned”* is deleted from the original definition based on generic considerations regarding procedures.

The word “permanent” is replaced with “persisting”, and a pre-defined timespan of 12 months is added to the word *“persisting”* based on general considerations regarding determining when a condition is to be classified as a Severe Toxicity. Renal failure requiring dialysis for ≥12 months is unlikely to resolve based on expert opinions.

Conditions fulfilling the clinical criteria are included whether they occur during treatment or after treatment. This decision was based on general considerations regarding treatment-related causality and the potential bias introduced by only including conditions emerging during cancer treatment.

## Pulmonary failure

| **Original ST definition** | **Additional notes** |
| --- | --- |
| Chronic lung failure (including pulmonary fibrosis and bronchiolitis obliterans) emerging during or after anticancer therapy and requiring daily oxygen supplement or lung transplantation (completed or planned) | - |

| **Modified ST definition** | **Additional notes** | **Time of ST** |
| --- | --- | --- |
| Chronic lung failure (including pulmonary fibrosis and bronchiolitis obliterans) emerging during or after anticancer therapy and requiring daily oxygen supplement (≥12 months) or lung transplantation. | - | Date when the condition fulfilling the clinical criteria in the ST definition has persisted for 12 months or when the patient is approved for transplantation (whichever occurs first). |

Changes and considerations:

A pre-defined timespan of 12 months is added based on general considerations regarding determining when a condition is to be classified as a Severe Toxicity. Bronchiolitis obliterans may occur after treatment cessation as part of cGVHD, increasing the importance of a pre-defined timespan. The original definition does theoretically allow for a severe infection requiring daily oxygen supplement to be included even if it only persists for a short period of time.

The phrase *“completed or planned”* is deleted from the original definition based on general considerations regarding procedures.

## Osteonecrosis

| **Original ST definition** | **Additional notes** |
| --- | --- |
| Osteonecrosis occurring during or after anticancer therapy and   - requiring total joint arthroplasty (completed or planned)   OR   - resulting in grade 4 toxicity according to the Ponte di Legno Toxicity Working Group Criteria (ie, symptomatic with deformation by imaging of one or more joints or substantially affecting self-care activity of daily living) at the time of STFS data capture. | - |

| **Modified ST definition** | **Additional notes** | **Time of ST** |
| --- | --- | --- |
| Osteonecrosis occurring during or after anticancer therapy and   - requiring total joint arthroplasty   OR   - resulting in grade 4 toxicity according to the Ponte di Legno Toxicity Working Group Criteria (i.e., symptomatic with deformation by imaging of one or more joints or substantially affecting self-care ADL* for ≥12 months)   *E.g., requiring daily assistance beyond what is considered age appropriate) with at least one self-care ADL and/or requiring instrumental aid, such as wheelchair or walking stick, for mobility. | Examples of self-care ADL include grooming/personal hygiene, dressing, toileting/continence, transferring/ambulating, and eating. | Date when the condition fulfilling the clinical criteria in the ST definition has persisted for 12 months or when total joint arthroplasty is performed (whichever occurs first). |

Changes and considerations:

A pre-defined timespan on 12 months is added for conditions that include evaluation of ADL based on general considerations on this topic.

The phrase *“completed or planned”* is deleted from the original definition based on general considerations regarding procedures. We acknowledge that there may be delay between when total joint arthroplasty is planned and when it is completed due to e.g., growth of the child, hence potentially affecting the timepoint where the osteonecrosis could be classified as an ST. However, we expect cases requiring surgery to also be captured as a grade 4 toxicity, thus allowing the classification of osteonecrosis as an ST to be as close to fulfillment of ST criteria as possible, despite not having undergone surgery yet.

The phrase “*at the time of STFS data capture*” is deleted since result should not depend on the time of data capture, which would also challenge the possibility to compare a retrospective study with future prospective studies.

Self-care ADL is used consistently for the physical conditions and examples are provided.

## Amputation and physical deformation

| **Original ST definition** | **Additional notes** |
| --- | --- |
| Amputation of extremities, severe spinal deformation, and disabling scleroderma, scarring, or contractions affecting self-care and instrumental ADL or causing substantial facial disfigurement and defined as follows:   - Lower limb amputation (proximal to ankle) - Upper limb amputation (proximal to wrist) - Scoliosis, kyphosis, or lordosis affecting ADL or requiring spinal surgery - Scarring or contractions affecting range of movement that affects ADL - Scleroderma caused by graft-versus-host disease affecting ADL - Amputation of nose - Amputation of one or both eyes - Complete facial palsy | Conditions are included if emerging during anticancer therapy. Scleroderma caused by chronic graft-versus-host disease and fulfilling the definition is included at any time point after hematopoietic stem-cell transplantation. |

| **Modified ST definition** | **Additional notes** | **Time of ST** |
| --- | --- | --- |
| Amputation of extremities, severe spinal deformation, and disabling scleroderma, scarring, or contractions affecting self-care ADL* substantially for ≥12 months or causing substantial facial disfigurement and defined as follows:   - Lower limb amputation (proximal to ankle) - Upper limb amputation (proximal to wrist) - Scoliosis, kyphosis, or lordosis affecting self-care ADL substantially - Scarring or contractions affecting range of movement that affects self-care ADL substantially - Scleroderma affecting self-care ADL substantially - Amputation of nose - Amputation of one or both eyes - Complete facial palsy (unilateral- or bilateral)   Conditions emerging during or after anticancer therapy are included. *E.g., requiring daily assistance beyond what is considered age-appropriate with at least one self-care ADL’s and/or requiring instrumental aid, such as wheelchair or walking stick, for mobility | Examples of self-care ADL include grooming/personal hygiene, dressing, toileting/continence, transferring/ambulating, and eating. | Date when amputation is performed, or when one of the following conditions fulfilling the clinical criteria in the ST definition has persisted for ≥12 months: scoliosis, kyphosis, lordosis, scarring or contractions, or scleroderma, or when diagnosed with complete facial palsy (unilateral or bilateral). |

Changes and considerations:

“*Instrumental ADL”* is deleted and only self-care ADL is used, since this definition include physical conditions (based on general considerations regarding evaluation of ADL). Examples are provided.

A pre-defined timespan on 12 months is added for conditions that include evaluation of ADL based on general considerations on this topic.

For spinal deformation the sentence “*requiring surgery”* is deleted since it is not considered unacceptable if the patient is no longer symptomatic after surgery. If the condition affects self-care ADL substantially and persists for ≥12 months, it is included.

The phrase “*completed or planned*” is deleted based on general considerations regarding procedures.

Both unilateral and bilateral complete facial palsy are included since both lead to substantially facial disfigurement.

Conditions fulfilling the clinical criteria are included whether they occur during treatment or after treatment. This decision was based on general considerations regarding treatment-related causality and the potential bias introduced by only including conditions emerging during cancer treatment. Furthermore, it was considered problematic if the inclusion of conditions (scleroderma) depends on which treatment the patient has received. This will lead to bias. It was decided to include all conditions occurring after anticancer therapy to avoid excluding important events caused by the treatment. Potentially including non-treatment-related amputations/deformations will not affect any comparison between treatment protocols. Furthermore, the conditions are rare, so the non-treatment-related cases are not expected to make any major differences in the outcome measure.

## Cognitive dysfunction

| **Original ST definition** | **Additional notes** |
| --- | --- |
| Any substantial impairment of neurocognitive functions (eg, executive function [planning and organization], sustained attention, memory [particularly visual sequencing, temporal memory], processing speed, visual-motor integration, fine motor dexterity, diminished performance on IQ-tests, and learning deficits) emerging during or after anticancer therapy, that severely restricts participation in school, vocational training, practice, career, or other key activities of instrumental ADL. | As evaluated by the physician, since uniform and objective neurocognitive evaluation is not done across study groups. |

| **Modified ST definition** | **Additional notes** | **Time of ST** |
| --- | --- | --- |
| Any substantial impairment of neurocognitive functions* emerging during or after anticancer therapy, that affects instrumental ADL** substantially and persists for ≥12 months after ending anticancer therapy.  *E.g., executive function (planning and organization), sustained attention, memory (particularly visual sequencing, temporal memory), processing speed, visual-motor integration, fine motor dexterity, diminished performance on IQ-tests, and learning deficits)  **E.g., severely restricted participation in school, vocational training, practice, and career, and/or requiring daily assistance beyond what is considered age-appropriate with other key activities of instrumental ADL | Cognitive dysfunction emerging after ending anticancer therapy must persist for ≥12 months to be classified as an ST. Pre-existing conditions relevant for this condition include not having met normal developmental milestones and/or evidence of developmental delay at the time of diagnosis. Other examples of instrumental ADL include cooking, cleaning, managing finances, managing medications.  “Verified” cognitive dysfunction is defined as: The patient lives in an institution due to cognitive dysfunction and/or the patient requires daily assistance with instrumental ADL due to cognitive dysfunction  “Possible” cognitive dysfunction is defined as: The patient scores below normal range (<2.5 percentile) in neuropsychological tests^§^, but the degree of impact on instrumental ADL is uncertain and/or the patient has substantially impaired instrumental ADL, but it is uncertain if it depends on this specific condition or is due to other reasons, e.g., psychiatric disease.  § Not specified due to variety in instruments used across centers. Neuropsychological testing is likely to be performed in patients with severe cognitive dysfunction but is not required for inclusion in the STFS measure. | Date when the condition fulfilling the clinical criteria in the ST definition has persisted for 12 months after ending anticancer therapy. |

Changes and considerations:

Instrumental ADL is used for cognitive/psychiatric conditions and examples are provided.

A pre-defined timespan is added for conditions that include evaluation of ADL based on general considerations on this topic. The duration of this condition was required to include ≥12 months after ending anticancer therapy, since it could strongly, but temporarily be affected by acute, temporary side effects like nausea, fatigue, and pain while on treatment, which may last for several years (e.g., 2-3 years for childhood ALL).

Relevant pre-existing conditions include not having met normal developmental milestone and/or evidence of developmental delay at time of diagnosis, which should be registered at data capture in alignment with the general decision to capture pre-existing conditions allowing these to be considered in the analyses.

Classification of this ST is challenging due to lack of standardized evaluation. Objective evaluation and neuropsychological testing are not done systematically and uniformly across study groups hence the classification of this toxicity is based on the evaluation by the reporting physician providing the STFS measure with some degree of subjectivity. Therefore, the outcome is divided into “Possible cognitive dysfunction” and “Verified cognitive dysfunction allowing the data analyses to consider a “worst case scenario” and a “best case scenario”, hereby addressing the uncertainty accompanying the classification of this ST.

“Verified cognitive dysfunction” was defined as:

- The patient lives in an institution due to cognitive dysfunction

OR

- The patient requires daily assistance with instrumental ADL due to cognitive dysfunction

“Possible cognitive dysfunction” was defined as:

- The patient scores below normal range (<2.5 percentile) in neuropsychological tests^§^, but the degree of impact on instrumental ADL is uncertain

AND/OR

- The patient has substantially impaired instrumental ADL, but it is uncertain if it depends on this specific condition or is due to other reasons, e.g., psychiatric disease.

^§^Not specified due to variety in instruments used across centers

## Seizures

| **Original ST definition** | **Additional notes** |
| --- | --- |
| Seizures emerging during anticancer therapy that require neurosurgical intervention (completed or planned) to reach control, or that fulfil the International League Against Epilepsy definition for drug-resistant epilepsy (namely the failure of adequate trials of two tolerated and appropriately chosen and used anti-epileptic drug schedules, whether as monotherapies or in combination, to reach sustained seizure freedom). | - |

| **Modified ST definition** | **Additional notes** | **Time of ST** |
| --- | --- | --- |
| Seizures emerging during or after anticancer therapy that require neurosurgical intervention to reach seizure control, or that fulfil the International League Against Epilepsy definition for drug-resistant epilepsy (”defined as failure of adequate trials of two tolerated and appropriately chosen and used anti-epileptic drug schedules (whether as monotherapies or in combination) to reach sustained seizure freedom”) (16). | - | Date when the patient experiences seizures despite adequate trials of two tolerated and appropriately chosen and used anti-epileptic drug schedules, or when neurosurgery is performed to reach seizure control (whichever occurs first) . |

Changes and considerations

The phrase “*completed or planned*” is deleted based on general considerations regarding procedures.

Conditions fulfilling the clinical criteria are included whether they occur during treatment or after treatment. This decision was based on general considerations regarding treatment-related causality and the potential bias introduced by only including conditions emerging during cancer treatment. Literature supports that treatment-related toxicities may lead to seizures emerging after anticancer therapy (17), e.g., cerebral cavernous malformations after cranial radiation (18,19).

The wording is changed slightly making the elaboration on drug-resistant epilepsy a direct quote from the International League Against Epilepsy (ILAE) definition (16).

It is acknowledged that drug responsiveness of a patient’s epilepsy is not a fixed state, i.e., it may change over time. It was discussed to require >1 seizure per year for classification as an ST, however, it was found most appropriate to simply use the ILAE definition for drug-resistant epilepsy. If the ILAE definition changes, the ST definition should be changed accordingly.

## Psychiatric disease

| **Original ST definition** | **Additional notes** |
| --- | --- |
| Any psychiatric or mental health disorder, emerging during anticancer therapy, that is severe enough to require ongoing mental health input (psychology or psychiatry), and is not adequately controlled ~~(~~ie, the condition severely restricts participation in school, vocational training, practice, or career, or other instrumental ADL) by medical, mental, or other therapeutic interventions. | As evaluated by the physician because uniform and objective evaluation is not across study group. Cases with any known psychiatric disease before acute lymphocytic leukaemia diagnosis are excluded. |

| **Modified ST definition** | **Additional notes** | **Time of ST** |
| --- | --- | --- |
| Any psychiatric disorder emerging during or after anticancer therapy, that is severe enough to require mental health input (psychology or psychiatry), and affects instrumental ADL substantially* and persists for ≥12 months after ending anticancer therapy.  *E.g., severely restricted participation in school, vocational training, practice, and career, and/or requiring daily assistance beyond what is considered age-appropriate with other key activities of instrumental ADL. | As evaluated by the physician because uniform and objective evaluation is not done across study groups.  Other examples of instrumental ADL include cooking, cleaning, managing finances, managing medications. | Date when the condition fulfilling the clinical criteria in the ST definition has persisted for 12 months after ending anticancer therapy. |

Changes and considerations

A pre-defined timespan is added for conditions that include evaluation of ADL based on general considerations on this topic. For cognitive dysfunction and psychiatric disorders, the timespan will be 12 months after treatment cessation since they may be severely but temporarily affected during treatment, and, in some patients, for a prolonged period hereafter. There is no evidence to support a specific cut-point after which it is unlikely that a psychiatric disorder will resolve. However, not many psychiatric diseases are chronic in children, except from examples as autism and schizophrenia. For some of the most common disorders, e.g., depression and anxiety, patients will often improve within 1 year with or without treatment, suggesting that 12 months is an appropriate time span in the opinion of experts.

Conditions fulfilling the clinical criteria are included whether they occur during treatment or after treatment. This decision was based on general considerations regarding treatment-related causality and the potential bias introduced by only including conditions emerging during cancer treatment. “*Mental health disorder”* is deleted since it is considered unspecific. If it leads to severe cognitive dysfunction, it will be classified as such.

In the phrase “*Require ongoing mental health input”* it is unclear what “*ongoing”* means and it cannot be applied in a prospective setting. *“Ongoing”* is therefore deleted and the classification of psychiatric disorder as an ST relies on evaluating instrumental ADL. Instrumental ADL is used for the cognitive/psychiatric conditions and examples are provided.

It cannot be known with certainty, that a condition is treatment related. The causality is especially challenging for conditions as psychiatric disorder, which also occurs in a significant proportion of the background population. However, some patients do become very affected by the anticancer treatment, and they should be included in this measure.

The note “*Patients with known psychiatric disease will be excluded”* is deleted since it would lead to bias if patients with known psychiatric disease were excluded from the start. However, it is highly relevant to register if the patient suffers from a known psychiatric disorder, allowing the analyses to take pre-existing conditions, that may increase the risk of developing a specific ST, into account. Hence, all known, relevant pre-existing conditions for any ST should be registered for each patient.

## Paralytic, myopathic and movement disorders

| **Original ST definition** | **Additional notes** |
| --- | --- |
| Paralytic, neuropathic (e.g., paraesthesia, numbness, or pain), myopathic (e.g., generalized muscle weakness caused by rhabdomyolysis) or movement disorders (e.g., ataxia) emerging during anticancer therapy that substantially affects ADL which includes impaired gait to a degree necessitating wheelchair or other instrumental aid or substantially impaired upper or lower limb function (i.e., severely restricting age-appropriate instrumental and self-care ADL). | - |

| **Modified ST definition** | **Additional notes** | **Time of ST** |
| --- | --- | --- |
| Paralytic, neuropathic (e.g., paresthesia, numbness, or pain), myopathic (e.g., generalized muscle weakness caused by rhabdomyolysis) or movement disorders (e.g., ataxia) emerging during or after anticancer therapy that substantially affects self-care ADL* for ≥12 months.  *e.g., requiring daily assistance beyond what is considered age-appropriate with at least one self-care ADL’s and/or requiring instrumental aid, such as wheelchair or walking stick, for mobility. | Examples of self-care ADL include grooming/personal hygiene, dressing, toileting/continence, transferring/ambulating, and eating. | Date when the condition fulfilling the clinical criteria in the ST definition has persisted for 12 months. |

Changes and considerations:

A pre-defined timespan on 12 months is added for conditions that include evaluation of ADL based on general considerations on this topic. Neuropathy is a common side effect (20), primarily associated with vincristine treatment. A significant proportion of patients with neuropathy symptoms will improve within 12 months, but in some patients the symptoms persist (20,21). If the condition fulfills the clinical criteria in the ST definition for ≥12 months, it is considered of such severity that it should be included as an ST irrespective of a potential recovery after 12 months.

Conditions fulfilling the clinical criteria are included whether they occur during treatment or after treatment. This decision was based on general considerations regarding treatment-related causality and the potential bias introduced by only including conditions emerging during cancer treatment. Self-care ADL is used for the physical conditions and examples are provided.

## Vocal cord paralysis

| **Original ST definition** | **Additional notes** |
| --- | --- |
| Permanent vocal cord paralysis, either unilateral or bilateral, emerging during anticancer therapy, requiring ventilatory support or tracheostomy, or leading to substantially reduced ability or inability to produce speech sounds. | - |

| **Modified ST definition** | **Additional notes** | **Time of ST** |
| --- | --- | --- |
| Persisting (≥12 months) vocal cord paralysis, either unilateral or bilateral, emerging during or after anticancer therapy, requiring ventilatory support (e.g., non-invasive ventilation) or tracheostomy, or leading to substantially reduced ability or inability to produce speech sounds. | - | Date when the condition fulfilling the clinical criteria in the ST definition has persisted for 12 months. |

Changes and considerations:

The word “permanent” is replaced with the word “persisting” and a pre-defined timespan of 12 months is added based on general considerations regarding determining when a condition is to be classified as a Severe Toxicity. Vocal cord paralysis persisting for ≥12 months is unlikely to resolve in the opinion of experts. It may not necessarily be mentioned in the medical charts if the condition is still present or not. This is a general limitation.

Conditions fulfilling the clinical criteria are included whether they occur during treatment or after treatment. This decision was based on general considerations regarding treatment-related causality and the potential bias introduced by only including conditions emerging during cancer treatment.An example of ventilatory support is provided (non-invasive ventilation) for clarification.

## Cytopenia

| **Original ST definition** | **Additional notes** |
| --- | --- |
| Profound and permanent cytopenia in one or more haematopoietic cell lines, without evidence of haematopoietic recovery, emerging during anticancer therapy and requiring HSCT (completed or planned) | Myelodysplastic syndromes are captured as second malignant neoplasms. Known underlying predisposing condition likely to explain the cytopenia is reported at time of severe toxicity data capture. |

| **Modified ST definition** | **Additional notes** | **Time of ST** |
| --- | --- | --- |
| Profound and permanent cytopenia in one or more haematopoietic cell lines, without evidence of haematopoietic recovery, emerging during or after anticancer therapy and requiring HSCT. | Myelodysplastic syndromes are captured as second malignant neoplasms. | Date when the patient is referred for HSCT due to cytopenia. |

Changes and considerations:

The phrase “*completed or planned”* is deleted based on general considerations regarding procedures.

Conditions fulfilling the clinical criteria are included whether they occur during treatment or after treatment. This decision was based on general considerations regarding treatment-related causality and the potential bias introduced by only including conditions emerging during cancer treatment.

The timepoint when the patient is referred for transplantation is used as the time of ST since that information is expected to be available, and there may be delay between planning and completing the procedure while waiting for a suitable donor.

The note “*Known underlying predisposing condition likely to explain the cytopenia is reported at time of severe toxicity data capture”* is deleted based on generic considerations regarding pre-existing and predisposing conditions.

Specific cell counts defining cytopenia is not necessary. The criteria that should be met for being classified as having this ST is that the cytopenia requires HSCT.

## Immunodeficiency

| **Original ST definition** | **Additional notes** |
| --- | --- |
| Permanent immunodeficiency emerging during anticancer therapy and requiring HSCT (completed or planned) | Cases with known underlying primary immune deficiency, identified at any timepoint before data capture are included and the underlying condition is reported at time of severe toxicity data capture. Severe leukopenia requiring HSCT is classed as cytopenia. |

| **Modified ST definition** | **Additional notes** | **Time of ST** |
| --- | --- | --- |
| Permanent immunodeficiency emerging during or after anticancer therapy and requiring HSCT. | Severe leukopenia requiring HSCT is classed as cytopenia. | Date when the patient is referred for HSCT due to immunodeficiency. |

Changes and considerations:

The phrase *“completed or planned”* is deleted based on general considerations regarding procedures.

The timepoint when the patient is referred for transplantation is used as the time of ST since that information is expected to be available, and there may be delay between planning and completing the procedure while waiting for a suitable donor.

Conditions fulfilling the clinical criteria are included whether they occur during treatment or after treatment. This decision was based on general considerations regarding treatment-related causality and the potential bias introduced by only including conditions emerging during cancer treatment.

The note *“Cases with known underlying primary immune deficiency, identified at any timepoint before data capture are included and the underlying condition is reported at time of severe toxicity data capture”* is deleted based on generic considerations regarding pre-existing and predisposing conditions.

## Second malignant neoplasms and benign central nervous system tumors

| **Original ST definition** | **Additional notes** |
| --- | --- |
| Second malignant neoplasms or benign CNS tumours emerging during or after anticancer therapy. | Non-melanoma skin cancers are not included. Known underlying cancer prone syndromes are reported at time of severe toxicity data capture. |

| **Modified ST definition** | **Additional notes** | **Time of ST** |
| --- | --- | --- |
| Second malignant neoplasms or benign central nervous system tumors emerging during or after anticancer therapy. | Non-melanoma skin cancers are not included. | Date when malignant neoplasm or benign central nervous system tumor is diagnosed. |

Changes and considerations:

The note “*Known underlying cancer prone syndromes are reported at time of severe toxicity data capture”* is deleted based on general discussions regarding pre-existing and predisposing conditions.

# Original and modified generic ST inclusion criteria

Through the Delphi process reviewing the modified ST definitions, it became clear that some of the problems identified with the original definitions also concerned two out of the five generic inclusion criteria. Based on plenary discussions, the Danish study group (including a biostatistician) proposed modification to the two concerned generic criteria, which were also reviewed in a Delphi process. Consensus was defined a priori as 100% consensus. Near-full consensus was reached already after 1^st^ round, with only two comments regarding minor changes in phrasing (available upon request). Full consensus was reached after two Delphi rounds.

Changes and underlying considerations

The first generic criterion “Not present before diagnosis of acute lymphocytic leukemia” needed to be revised. When evaluating treatment-related toxicities it is intuitive to only include conditions that were not present before cancer diagnosis. However, patients may suffer from pre-existing conditions that may affect the risk of developing certain STs. For each toxicity, all relevant pre-existing conditions must be registered to allow for valid statistical analyses. Relevant pre-existing conditions may either increase the probability of having a severe toxicity classified (e.g., being blind in one eye so only the other eye needs to be affected during treatment; or having Li-Fraumeni syndrome, relevant for developing second malignant cancer), or preclude the possibility of a classification as severe toxicity for the patient (e.g., being blind in both eyes before cancer diagnosis). Excluding all patients with any of these conditions from the start may be a problem for composite outcomes like STFS or the burden of multiple toxicities since this would lead to a selected population. On the other hand, including the patient that can never be classified as having a specific toxicity will introduce bias towards a lower burden of STs and/or potential confounding of comparisons of treatment protocols. Hence, it was necessary to address this issue in the generic criterion.

The last generic criterion *“Permanent or correctable only by an unacceptable treatment”* was necessary to revise due to the general problem with the use of the term “*permanent”*. To make valid statistical analyses of time-to-event data it is important to not condition on the future (22), therefore, the 'time of the severe toxicity' must be the time where the toxicity can be identified/classified as a 'severe toxicity', and not a retrospective assessment. The word “*permanent”* has been replaced with word “*persisting”* and the concerned definitions are furthermore provided with a pre-defined timespan at the end of which the condition is classified as a Severe Toxicity. This decision should also be mirrored in the generic criterion, where the word “*permanent”* is replaced with the word “*persisting”.*

| **Original generic criteria** |
| --- |
| **Not present before diagnosis of acute lymphocytic leukemia**   - Not present before the cancer diagnosis; only conditions occurring during or after cancer diagnosis are included |
| **Symptomatic**   - To ensure equal probability of capturing the condition across different protocols using different screening strategies, the condition must be symptomatic and expected to lead to a clinical diagnosis without use of routine screening. - Compensated cardiac failure detected by routine echocardiogram is not included, whereas severe, symptomatic cardiac failure is included. |
| **Objective**   - The condition must be uniformly classifiable across different patients and by different observers. - Chronic pain, nausea, or fatigue, which are subjective, are not included, although these conditions can represent a substantial burden to the survivor. |
| **Unacceptable severity**   - The condition must be so severe, that it is considered an unacceptable tradeoff for disease control—ie, had the condition been predictable at acute lymphocytic leukaemia diagnosis, it would probably have led to a change in anticancer therapy. - Physical and mental conditions that substantially affect self-care and instrumental activities of daily living or posing substantial threat of early mortality fulfil this criterion. - This consideration mirrors current actions (eg, as reduction of anthracycline use in patients with Down Syndrome, reduction of thiopurine doses in patients with TPMT deficiency) or concerns related to re-exposure after severe drug-induced toxicity (eg, re-exposure to asparaginase following asparaginase associated pancreatitis). |
| **Permanent or correctable only by unacceptable treatments**   - The condition must be anticipated to be permanent and present at severe toxicity capture have been corrected by a treatment, which in itself is considered unacceptable—ie, radical and invasive, as specified in the individual definitions - Acute events are not included, but sequelae such as severe cognitive deficits following cerebral haemorrhage or amputation of a limb following severe infections, are. - Organ transplantation is an example of an unacceptable treatment since it is itself associated with risk of severe mortality and morbidity, whereas growth hormone replacement is not considered an unacceptable treatment. |

| **Modified generic criteria** |
| --- |
| **Not present before cancer diagnosis**  Pre-existing conditions cannot be considered treatment-related toxicity. For each toxicity, all relevant pre-existing conditions must be registered to allow for valid statistical analyses. Relevant pre-existing conditions may either   - increase the probability of having a severe toxicity classified (e.g., being blind in one eye so only the other eye needs to be affected during treatment; or having Li-Fraumeni syndrome, relevant for developing second malignant cancer), or - preclude the possibility of a classification as severe toxicity for the patient (e.g., being blind in both eyes before cancer diagnosis) |
| **Symptomatic**  To ensure equal probability of capturing the condition across different protocols using different screening strategies, the condition must be symptomatic and expected to lead to a clinical diagnosis without use of routine screening.   - Compensated cardiac failure detected by routine echocardiogram is not included, whereas severe, symptomatic cardiac failure is included. |
| **Objective**  The condition must be uniformly classifiable across different patients and by different observers.   - Chronic pain, nausea, or fatigue, which are subjective, are not included, although these conditions can represent a substantial burden to the survivor. |
| **Unacceptable severity**  The condition must be so severe, that it is considered an unacceptable tradeoff for disease control—i.e., had the condition been predictable at cancer diagnosis, it would probably have led to a change in anticancer therapy.   - Physical and mental conditions that substantially affect self-care and instrumental activities of daily living or posing substantial threat of early mortality fulfil this criterion. - This consideration mirrors current actions (e.g., as reduction of anthracycline use in patients with Down Syndrome, reduction of thiopurine doses in patients with TPMT deficiency) or concerns related to re-exposure after severe drug-induced toxicity (e.g., re-exposure to asparaginase following asparaginase associated pancreatitis). |
| **Persisting severity or correctable only by unacceptable treatments**  The condition must have been present for a sustained period or be corrected by a treatment, which itself is considered unacceptable.   - Acute events are not included, but sequelae such as severe cognitive deficits following cerebral hemorrhage or amputation of a limb following severe infections, are. - Organ transplantation is an example of an unacceptable treatment since it is itself associated with risk of severe mortality and morbidity, whereas growth hormone replacement is not considered an unacceptable treatment. |

# Reference list

1. Pui CH, Schrappe M, Masera G, Nachman J, Gadner H, Eden OB, et al. Ponte di Legno working group: Statement on the right of children with leukemia to have full access to essential treatment and report on the Sixth International Chilhood Acute Lymphoblastic Leukemia Workshop. Leukemia. 2004;18(6):1043–53.

2. Andrés-Jensen L, Attarbaschi A, Bardi E, Barzilai-Birenboim S, Bhojwani D, Hagleitner MM, et al. Severe toxicity free survival: physician-derived definitions of unacceptable long-term toxicities following acute lymphocytic leukaemia. Lancet Haematol. 2021 Jul;8(7):e513–23.

3. Boulkedid R, Abdoul H, Loustau M, Sibony O, Alberti C. Using and reporting the Delphi method for selecting healthcare quality indicators: a systematic review. PLoS One. 2011;6(6):e20476.

4. Diamond IR, Grant RC, Feldman BM, Pencharz PB, Ling SC, Moore AM, et al. Defining consensus: a systematic review recommends methodologic criteria for reporting of Delphi studies. J Clin Epidemiol. 2014 Apr;67(4):401–9.

5. Fetoni AR, Brigato F, De Corso E, Lucidi D, Sergi B, Scarano E, et al. Long-term auditory follow-up in the management of pediatric platinum-induced ototoxicity. Eur Arch oto-rhino-laryngology Off J Eur Fed Oto-Rhino-Laryngological Soc Affil with Ger Soc Oto-Rhino-Laryngology - Head Neck Surg. 2022 Oct;279(10):4677–86.

6. Grewal S, Merchant T, Reymond R, McInerney M, Hodge C, Shearer P. Auditory late effects of childhood cancer therapy: a report from the Children’s Oncology Group. Pediatrics. 2010 Apr;125(4):e938-50.

7. Bertolini P, Lassalle M, Mercier G, Raquin MA, Izzi G, Corradini N, et al. Platinum compound-related ototoxicity in children: long-term follow-up reveals continuous worsening of hearing loss. J Pediatr Hematol Oncol. 2004 Oct;26(10):649–55.

8. Bass JK, Hua C-H, Huang J, Onar-Thomas A, Ness KK, Jones S, et al. Hearing Loss in Patients Who Received Cranial Radiation Therapy for Childhood Cancer. J Clin Oncol Off J Am Soc Clin Oncol. 2016 Apr;34(11):1248–55.

9. Romano A, Capozza MA, Mastrangelo S, Maurizi P, Triarico S, Rolesi R, et al. Assessment and Management of Platinum-Related Ototoxicity in Children Treated for Cancer. Cancers (Basel). 2020 May;12(5).

10. Bansal N, Amdani S, Lipshultz ER, Lipshultz SE. Chemotherapy-induced cardiotoxicity in children. Expert Opin Drug Metab Toxicol. 2017;13(8):817–32.

11. Leerink JM, de Baat EC, Feijen EAM, Bellersen L, van Dalen EC, Grotenhuis HB, et al. Cardiac Disease in Childhood Cancer Survivors: Risk Prediction, Prevention, and Surveillance: JACC CardioOncology State-of-the-Art Review. JACC CardioOncology. 2020 Sep;2(3):363–78.

12. Silverman LB. Balancing cure and long-term risks in acute lymphoblastic leukemia. Hematology. 2014;2014(1):191–7.

13. Lipshultz SE, Lipsitz SR, Sallan SE, Dalton VM, Mone SM, Gelber RD, et al. Chronic progressive cardiac dysfunction years after doxorubicin therapy for childhood acute lymphoblastic leukemia. J Clin Oncol. 2005;23(12):2629–36.

14. Armstrong GT, Joshi VM, Ness KK, Marwick TH, Zhang N, Srivastava D, et al. Comprehensive Echocardiographic Detection of Treatment-Related Cardiac Dysfunction in Adult Survivors of Childhood Cancer: Results From the St. Jude Lifetime Cohort Study. J Am Coll Cardiol. 2015 Jun;65(23):2511–22.

15. Wolthers BO, Frandsen TL, Baruchel A, Attarbaschi A, Barzilai S, Colombini A, et al. Asparaginase-associated pancreatitis in childhood acute lymphoblastic leukaemia: An observational Ponte di Legno Toxicity Working Group study. Lancet Oncol. 2017;2045(17):1–11.

16. Kwan P, Arzimanoglou A, Berg AT, Brodie MJ, Allen Hauser W, Mathern G, et al. Definition of drug resistant epilepsy: consensus proposal by the ad hoc Task Force of the ILAE Commission on Therapeutic Strategies. Epilepsia. 2010 Jun;51(6):1069–77.

17. Goldsby RE, Liu Q, Nathan PC, Bowers DC, Yeaton-Massey A, Raber SH, et al. Late-occurring neurologic sequelae in adult survivors of childhood acute lymphoblastic leukemia: a report from the Childhood Cancer Survivor Study. J Clin Oncol Off J Am Soc Clin Oncol. 2010 Jan;28(2):324–31.

18. Singla A, Brace O’Neill JE, Smith E, Scott RM. Cavernous malformations of the brain after treatment for acute lymphocytic leukemia: presentation and long-term follow-up. J Neurosurg Pediatr. 2013 Feb;11(2):127–32.

19. Menon RN, Baheti NN, Cherian A, Rathore C, Iyer RS, Radhakrishnan A. Post-irradiation “acquired cavernous angiomas” with drug resistant seizures. Epilepsy Res. 2011 Sep;96(1–2):161–5.

20. Rodwin RL, Kairalla JA, Hibbitts E, Devidas M, Whitley MK, Mohrmann CE, et al. Persistence of chemotherapy-induced peripheral neuropathy despite vincristine reduction in childhood b-acute lymphoblastic leukemia. J Natl Cancer Inst. 2022 May;

21. Goodenough CG, Diouf B, Yang W, Sapkota Y, Finch ER, Lu L, et al. Association between CEP72 genotype and persistent neuropathy in survivors of childhood acute lymphoblastic leukemia. Leukemia. 2022 Apr;36(4):1160–3.

22. Andersen PK, Keiding N. Interpretability and importance of functionals in competing risks and multistate models. Stat Med. 2012 May;31(11–12):1074–88.

**
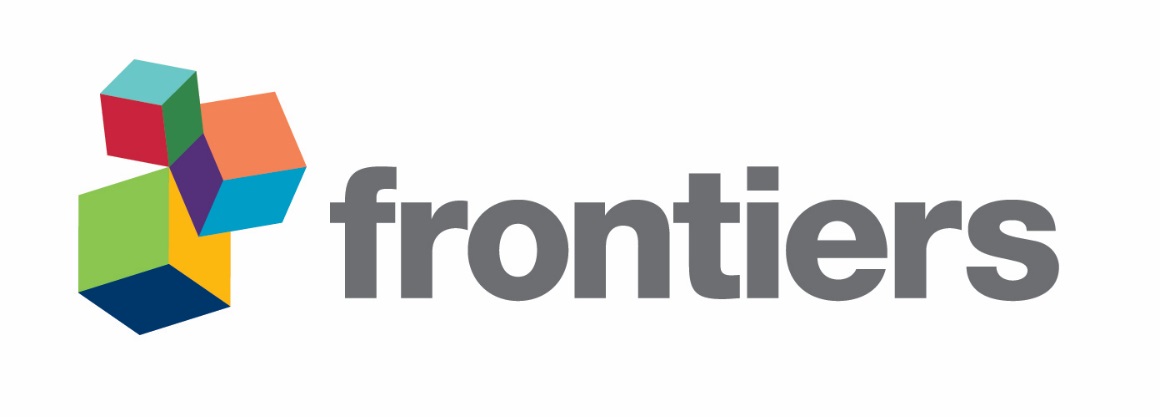
**
